# A multicenter cluster randomized, stepped wedge implementation trial for targeted normoxia in critically ill trauma patients: study protocol and statistical analysis plan for the Strategy to Avoid Excessive Oxygen (SAVE-O2) trial

**DOI:** 10.1186/s13063-021-05688-6

**Published:** 2021-11-08

**Authors:** Layne Dylla, David J. Douin, Erin L. Anderson, John D. Rice, Conner L. Jackson, Vikhyat S. Bebarta, Christopher J. Lindsell, Alex C. Cheng, Steven G. Schauer, Adit A. Ginde

**Affiliations:** 1 Department of Emergency Medicine, University of Colorado School of Medicine, Aurora, CO USA; 2 Department of Anesthesiology, University of Colorado School of Medicine, Aurora, CO USA; 3 Department of Biostatistics and Informatics, Colorado School of Public Health, Aurora, CO USA; 4 US Air Force 59th Medical Wing, Office of the Chief Scientist, JBSA, Lackland, San Antonio, TX USA; 5 Center for COMBAT Research, Department of Emergency Medicine, University of Colorado School of Medicine, Aurora, CO USA; 6 Department of Biostatistics, Vanderbilt University Medical Center, Nashville, TN USA; 7 Department of Biomedical Informatics, Vanderbilt University Medical Center, Nashville, TN USA; 8 US Army Institute of Surgical Research, JBSA Fort Sam, Houston, TX USA; 9 Department of Emergency Medicine, Brooke Army Medical Center, San Antonio, TX USA

**Keywords:** Oxygenation, Hyperoxia, Trauma, Injuries, Critical care, Intensive care units

## Abstract

**Background:**

Targeted normoxia (SpO_2_ 90–96% or PaO_2_ 60–100 mmHg) may help to conserve oxygen and improve outcomes in critically ill patients by avoiding potentially harmful hyperoxia. However, the role of normoxia for critically ill trauma patients remains uncertain. The objective of this study is to describe the study protocol and statistical analysis plan for the Strategy to Avoid Excessive Oxygen for Critically Ill Trauma Patients (SAVE-O2) clinical trial.

**Methods:**

Design, setting, and participants: Protocol for a multicenter cluster randomized, stepped wedge implementation trial evaluating the effectiveness of a multimodal intervention to target normoxia in critically ill trauma patients at eight level 1 trauma centers in the USA. Each hospital will contribute pre-implementation (control) and post-implementation (intervention) data. All sites will begin in the control phase with usual care. When sites reach their randomly assigned time to transition, there will be a one-month training period, which does not contribute to data collection. Following the 1-month training period, the site will remain in the intervention phase for the duration of the trial.

Main outcome measures: The primary outcome will be supplemental oxygen-free days, defined as the number of days alive and not on supplemental oxygen. Secondary outcomes include in-hospital mortality to day 90, hospital-free days to day 90, ventilator-free days (VFD) to day 28, time to room air, Glasgow Outcome Score (GOS), and duration of time receiving supplemental oxygen.

**Discussion:**

SAVE-O2 will determine if a multimodal intervention to improve compliance with targeted normoxia will safely reduce the need for concentrated oxygen for critically injured trauma patients. These data will inform military stakeholders regarding oxygen requirements for critically injured warfighters, while reducing logistical burden in prolonged combat casualty care.

**Trial registration:**

ClinicalTrials.govNCT04534959. Registered September 1, 2020.

**Supplementary Information:**

The online version contains supplementary material available at 10.1186/s13063-021-05688-6.

## Background

Oxygen therapy has undisputed importance in the care of critically ill patients to prevent morbidity associated with hypoxia and enhance oxygen delivery [1, 2]. Excessive oxygen supplementation to critically ill patients, resulting in hyperoxia, appears to be routine [3, 4] and may even be harmful [3, 5–8]. This practice also has critical consequences in terms of logistics during military operations, particularly in prolonged field care settings. A recent systematic review of 43 studies of oxygenation in critically ill patients identified few trauma-specific studies and none of high quality [8]. Therefore, there is ongoing uncertainty regarding the optimal use of oxygen therapy in critically ill trauma patients. Well-designed, trauma-specific trials are needed to guide oxygen targets in these patients.

To address this uncertainty, we are conducting the Strategy to Avoid Excessive Oxygen (SAVE-O2) for Critically Ill Trauma Patients clinical trial. The purpose of this trial is to determine the effectiveness of a multimodal educational intervention to safely reduce supplemental oxygen use in critically injured patients. Investigators will also evaluate the clinical effectiveness of the more targeted use of oxygen therapy. The pilot study for the SAVE-O2 trial demonstrated that the multimodal intervention is feasible and effective in reducing use of supplemental oxygen for critically ill trauma patients [9]. We convened an expert panel to develop consensus targets for oxygen saturation (SpO_2_), which determined optimal range of 90–96%, partial pressure of arterial oxygen (PaO_2_) range of 60–100 mmHg (when applicable), and fraction of inspired oxygen (FiO_2_) of 0.21 for mechanically ventilated patients or room air for non-mechanically ventilated patients [10, 11]. These ranges will be utilized to titrate supplemental oxygen use. Here we describe the protocol and statistical analysis plan for SAVE-O2. By using a pre-specified statistical analysis plan, we aim to reduce the risk of bias arising from knowledge of study findings as they emerge during data analyses [12].

## Methods

### Trial design

**Fig. 1:**
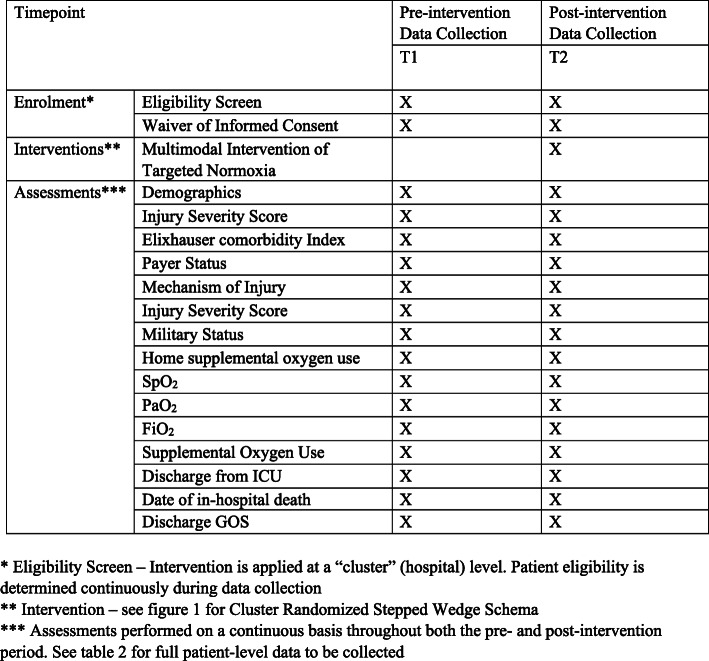
SPIRIT diagram

SAVE-O2 is a multicenter cluster randomized, stepped wedge implementation trial to evaluate the superiority of a multimodal educational intervention to improve adherence to consensus-based normoxia compared to usual care in critically ill trauma patients. This pragmatic design allows for optimal evaluation of an intervention where benefits of targeted normoxia outweigh the alternative of potential increased frequency of hypoxia and/or hyperoxia [13]. Eight level 1 trauma centers will be randomized over time to crossover from a pre-intervention (control) phase of usual care to post-intervention phase (targeted normoxia). Intervention at the hospital level minimizes the potential from contamination between participants in the pre- versus post-intervention stage. We detail the SAVE-O2 study design here, with reference to the Standard Protocol Items: Recommendations for Interventional Trials checklist [14] (Fig. 1 and Appendix 1). University of Colorado serves as the Clinical Coordinating Center (CCC) for this trial.

### Setting/Population

SAVE-O2 includes critically ill trauma patients from eight level 1 trauma centers geographically distributed throughout the United States. These hospitals have endorsed the consensus-based recommendation for normoxia (SpO_2_ 90–96%, PaO_2_ 60–100 mmHg) in critically ill trauma patients, but do not have existing protocols and/or resources to promote this oxygenation strategy. The target population includes adults aged 18 years or older who meet criteria for entry into state or national trauma registries and who are admitted to a surgical or trauma intensive care unit (ICU) within 24 h of arrival to a participating hospital. This includes patients who present directly to the emergency department of a participating hospital and those who are transferred into a participating hospital. Exclusion criteria include prisoners and known pregnancy. There is no selection for inclusion/exclusion based on mechanical ventilation status, injury severity, injury mechanism, or traumatic brain injury.

### Ethics approval

SAVE-O2 has been approved with a waiver of informed consent by the Colorado Multiple Institutional Review Board (COMIRB #19-2153), which serves as the single IRB for this study. Each enrolling site has ceded review to COMIRB under reliance agreements. Prior to the design of this study, we conducted a systematic review, Delphi consensus process, and pilot study. Together, these processes demonstrated preliminary safety evidence of targeted normoxia, identified consensus-based oxygenation targets, and provided a foundation to conduct this multicenter clinical trial with a waiver of informed consent under the Common Rule (45 CFR 46) that governs the ethical conduct and oversight of human subjects’ research, section 116 (d). The COMIRB determined that this trial represents no more than minimal risk to participants – this multimodal educational intervention helps hospitals implement a consensus-based oxygenation target that is not binding and remains at the discretion of the treating physician to ensure optimal care of all critically ill trauma patients. The use of a waiver of consent does not adversely affect the rights and welfare of subjects—patient care remains at the discretion of the clinical team to act in the best interests of an individual patient and data collection occurs at a hospital unit-level to ensure that patient care and privacy is not compromised. Finally, the study conducts research that could not practicably be carried out without the waiver of consent—there is no interaction with patients or their surrogates as the intervention is conducted at the level of each hospital unit with subjects as a whole receiving either usual care in the pre-intervention period or education-enhanced usual care in the post-intervention phase. The full details of the ethical considerations of this trial are detailed in the full study protocol (Appendix 2).

### Randomization

**Fig. 2:**
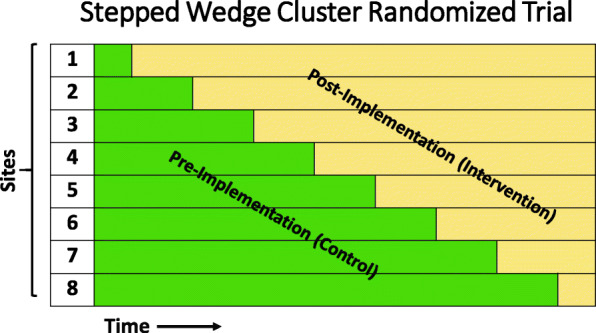
Schematic of multicenter cluster randomized, stepped wedge design. An overview of the multicenter cluster randomized, stepped wedge implementation design. Each “step” represents the crossover at 3-month intervals from the pre-implementation period to the post-implementation period, with the first month of the crossover period considered a wash-out period

Randomization occurs at the hospital level (“cluster”). In randomly chosen order, each cluster will cross over from the pre-intervention phase of usual care to post-intervention targeted normoxia. This incorporates a 1-month run-in period during which the hospital engages staff in educational activities and trainings designed to increase familiarity and compliance with targeted normoxia protocols. These standardized activities and associated materials are part of the “intervention” provided by the clinical coordinating center. During this run-in period, patient-level data collected will not be used for the primary analysis. Cluster crossover occurs every 3 months for a total study duration of 27 months (Fig. 2).

### Study Intervention

This trial evaluates the effectiveness of a multimodal educational intervention to increase compliance with targeted normoxia in critically ill trauma patients. Normoxia is defined based on a modified Delphi consensus approach with 31 nationally recognized military and civilian experts from trauma surgery, emergency medicine, critical care, and military operational medicine as a SpO_2_ of 90–96% or a PaO_2_ of 60–100 mmHg. This expert consensus panel defined the following oxygenation ranges based on non-invasive SpO_2_: hyperoxia as an SpO_2_ of greater than 96%, normoxia as an SpO_2_ of 90–96%, borderline as an SpO_2_ of 88–89%m and hypoxia as an SpO_2_ less than 88% [10, 11].

We take a multi-faceted approach to clinician and staff education at each site, starting with standardized educational presentations tailored to specific provider groups—physicians, nurses, and respiratory therapists. These presentations will (1) provide essential background information and stress the importance of avoiding both hypoxia and hyperoxia as a potential way to decrease patient morbidity and mortality, (2) give an overview of the SAVE-O2 trial, (3) provide potential protocols to down-titrate supplemental oxygen in hyperoxic patients, and (4) give instruction for reporting potential adverse events. In addition to these presentations, flyers will be placed strategically throughout sites reminding providers of the ongoing SAVE-O2 trial. Further education and reminders, led by local coordinators and investigators, will be conducted at staff meetings, floor rounds, and daily team meetings.

We will also provide monthly newsletters and feedback highlighting the pre- and ongoing post-intervention oxygenation practices. These will help guide sites in evaluating their progress toward a minimum goal of 90% of patient-hours spent within the targeted normoxia thresholds.

**Fig. 3:**
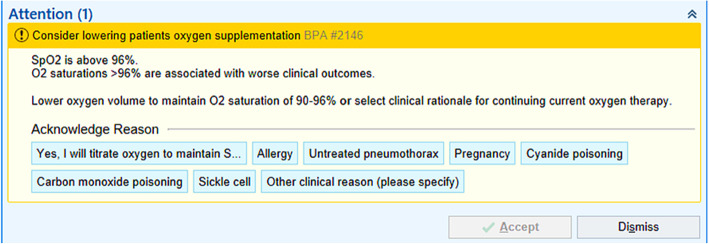
Electronic health record alert

In addition to multiple educational activities, some sites will also include an electronic health record best practice alert (Fig. 3). These alerts are designed in accordance with local practices, but generally alert nurses and respiratory therapists when a patient falls in the hyperoxia range based on SpO_2_ or PaO_2_ measurements. They will make recommendations based on on-site practice patterns for oxygen titration which can be overridden by clinical judgments. In cases where a recommendation for down-titration of supplementation oxygen is overridden by clinical judgment, that patient remains in the trial. The educational component of this intervention is expansive and targets physicians, advanced practice providers, nurses, and respiratory therapists. However, in many cases, nurses and respiratory therapists will be primarily responsible for oxygen titration. Each site is free to follow its own practices for specific oxygen titration and the exact time frame in which titrations should be made. However, SAVE-O2 recommends down titration of supplemental oxygen in patients with sustained hyperoxia (30 minutes or greater) by FiO_2_ increments of 0.1 until normoxia is reached, a mechanically ventilated patient is on an FiO_2_ of 0.21 or until a non-mechanically ventilated patient is no longer on supplemental oxygen. In some cases, oxygen titration may not be possible, for example, patients who are hyperoxic with an SpO_2_ > 96% but not on supplemental oxygen and those who are hypoxic but on an FiO_2_ of 1.0. Treating physicians are also allowed to override study recommendations when determined to be in the best interest of the patients. Potential situations where it is anticipated that treating physicians may temporarily favor hyperoxia include carbon monoxide poisoning, untreated pneumothorax, and cyanide poisoning.

Oxygen titration is encouraged from the time that a patient enters the hospital through to his/her discharge from the ICU. While SAVE-O2 outcomes focus on oxygenation in the ICU, the intervention will also target emergency department providers who often establish post-acute injury therapeutic momentum. To further increase the success of the intervention, leadership from within each site’s emergency department, trauma surgery teams, and the ICUs will work with study “champions” to reinforce intervention compliance. Oxygen titration is “encouraged” but not mandated because the primary study question focuses on the effectiveness of a multi-faceted educational intervention to target normoxia in critically ill trauma patients. Thus, we focus on educating ICU providers and assess the effect of the education intervention on the ICU oxygenation of these patients. After a patient is discharged from the ICU or transferred to a floor bed, the patient’s oxygenation status is no longer monitored for the purposes of this trial. Patients subsequently readmitted to the ICU from the floor after 24 h may still experience the effects of the educational intervention which is deployed at the level of the ICU unit, but no further oxygenation data is collected for that patient during the ICU readmission.

### Outcomes

#### Primary outcome

The primary outcome of SAVE-O2 is supplemental oxygen-free days (SOFD). This is defined as the number of days a patient is alive and not on supplemental oxygen from the time of presentation to day 28. SOFD is censored at hospital discharge. The score ranges from − 1 days (death) to 28 days (no supplemental oxygen use). Patients discharged from the hospital prior to day 28 are assumed to be maintained at the level of oxygenation they require on hospital discharge—i.e., if they are discharged on room air or the level of supplemental oxygen used prior to admission, it is assumed that they remained on no additional supplemental oxygen to day 28. When a patient receives the same level of supplemental oxygen as they require at home prior to admission, this will count as being free of supplemental oxygen. We will not count toward SOFD the amount of time patients are intubated and ventilated only for a surgical procedure and are immediately extubated upon completion of that procedure.

#### Secondary outcomes

There is no patient follow-up after hospital discharge in SAVE-O2 and all secondary outcomes are assessed at hospital discharge. However, variables will be censored at various intervals while hospitalized: hospital discharge, 28 days, or 90 days. SAVE-O2 will assess ventilator-free days to day 28 (VFD28) [10, 11, 15] as a secondary outcome. VFD28 is defined as 28 minus the number of days a patient is mechanically ventilated, censored at hospital discharge. Patients discharged on mechanical ventilation prior to 28 days are assumed to be maintained on mechanical ventilation to day 28 and will receive a score of zero if mechanical ventilation was initiated upon arrival. In cases of a failed extubation (reintubation in less than 48 h), this interval will count toward the number of days being ventilated. However, periods of mechanical ventilation lasting less than 24 h to facilitate surgical procedures or for sleep disordered breathing will not be counted toward ventilation days. Additionally, subjects who die prior to 28 days will be assigned a VFD28 score of − 1.

Secondary outcomes censored at 90 days include hospital-free days (HFD90), in-hospital mortality, and time to mortality. Hospital-free days is defined as the number of days a patient is alive and not hospitalized. The scores will range for − 1 days (worse outcome, patients who die in the hospital during the 90 days) or zero (patients who are hospitalized for more than 90 days) to 90 days (best outcome, patients who are discharged from the hospital alive to any location on the day of admission). In-hospital mortality is defined as alive or dead on the day of hospital discharge or day 90, whichever is first. Time to mortality is defined as the number of days from admission to death. Time to mortality is also censored at hospital discharge or day 90, whichever is first.

To better characterize oxygenation practices, we will collect data on multiple additional secondary outcomes. This includes the frequency and duration of ICU time a patient spends in the various oxygenation categories (hypoxia, borderline, normoxia, and hyperoxia) and the time spent compliant with targeted normoxia. For cases where a hyperoxic patient is ventilated on FiO_2_ of 0.21 or is on room air, no further oxygenation adjustments can be made. This time will count toward compliance with the normoxia protocol. We will also assess the time to the first incidence of no supplemental oxygen use (room air in non-mechanically ventilated patients or FIO_2_ of 0.21 in mechanically ventilated patients), censored at hospital discharge.

To characterize the potential reduction in the amount of supplemental oxygen used we will determine the total amount of supplemental oxygen administered. We will specifically examine the use of high levels of supplemental oxygen, defined as an FiO_2_ > 0.40 or more than 4 liters per minute. The proportion of participants on high levels of supplemental oxygen for more than 2 h in the ICU and the total duration of time receiving high levels of supplemental oxygen will be calculated.

The final patient-centered clinical outcomes include the discharge disposition and Glasgow Outcome Score (GOS) [16, 17]. Discharge disposition includes the following categories: expired, home, facility (i.e. long term care, rehabilitation facility, hospice, skilled nursing facility), long-term acute care facility, other (i.e., left against medical advice, missing). The GOS is a five-point scale rating the relative disability of patients from death to no disability that research staff will assign to a patient based on chart review. Limited independence due to orthopedic injuries and non-weight bearing status while fractures are healing will not count toward disability on the GOS scale.

### Data collection and management

SAVE-O2 will leverage the expertise of the Data Coordinating Center (DCC) at Vanderbilt University Medical Center and the on-site informatics specialists to implement protocols that automatically extract variables directly from the electronic health record and state trauma registry data (Table 1), including all recorded SpO_2_, PaO_2_, FiO_2_, and oxygen volume measurements. Many sites will also collect raw, unvalidated continuous SpO_2_ measurements. Instances that require manual chart extraction by site coordinators (i.e., GOS, home supplemental oxygen use, and military status) will be minimized and obtained using standardized operating procedures with flow charts and detailed procedures for extraction.

**Table 1:** Patient-level data collected

Source (extraction method)	Data collected
Trauma registry (automated extraction)	Date of presentation to the emergency department or hospital
	Date of ICU admission
	Age on day of admission
	Gender
	Race and ethnicity
	Payer status
	Elixhauser comorbidity index
	Mechanism of injury
	Injury Severity Score
Electronic health record (automated extraction)	Cigarette smoking status
	Body mass index
	Covid-19 status
	Shock Index
	All validated SpO_2_ values in ICU
	Unvalidated SpO_2_ values (continuous, recorded up to every minute when available) in ICU
	All PaO_2_ values in ICU
	All FiO_2_ values in ICU
	All PEEP values in ICU
	All oxygen volume measurements in ICU
	Date of discharge from ICU
	Date of in-hospital death
	Discharge disposition
Electronic health record (manual extraction)	Military status
	Home supplemental oxygen use
	Discharge GOS
Calculated outcomes	Supplemental Oxygen Free Day (SOFD) to day 28
	Ventilator-free days to day 28 (VFD28)
	Hospital-free days to day 90 (HFD90)
	In-hospital mortality to day 90
	Time to mortality to day 90
	Time to room air (or FiO_2_=0.21)
	Frequency of hypoxic episodes
	Duration of hypoxic episode
	Frequency of hyperoxic episodes
	Duration of hyperoxic episodes
	Total duration of time on normoxia protocol
	Amount of supplemental oxygen administered (total estimated oxygen volume administered in ICU)
	Duration of time on normoxia protocol target (time with SpO_2_ 90-96% or receiving no supplemental oxygen while in ICU)
	Proportion of participants receiving high levels of supplemental oxygen (FiO_2_ > 0.4 or more than 4LPM for more than 2 h while in ICU; excluding operating room time)
	Duration of time receiving high levels of supplemental oxygen
	Duration of time receiving no supplemental oxygen or FiO_2_ = 0.21

Abbreviations: *COVID-19* coronavirus disease 2019, *FiO*_*2*_ fraction of inspired oxygen, *GOS* Glasgow Outcome Scale, *ICU* intensive care unit; *LPM* liters per minute; *PaO*_*2*_ partial pressure of arterial oxygen, *PEEP* positive end expiratory pressure, *SpO*_*2*_ saturation of oxygen

SAVE-O2 uses the resources available at the DCC to provide specialized REDCap database development and management. REDCap is a secure, encrypted, HIPAA-compliant web application specifically designed for research data management with the ability for data capture and validation and data audits, and de-identified data export to common statistical packages [18]. Automated data extraction will take advantage of REDCap functionality that pulls data using electronic health record systems’ application programming interfaces (API) that conform to the HL7 Fast Health Interoperability Resources (FHIR) standard [19]. Additional accuracy and consistency checks will be performed by trained project managers at each site. Once data have been uploaded into a site’s REDCap database, REDCap deidentifies the data and sends it weekly to the DCC. The DCC assists with the automated feedback reports and shares these with the clinical coordinating center biostatistical core. Protected health information will only be accessible by the local site and not shared with other sites, the DCC or the CCC. Sites will be intermittently audited by the clinical coordinating center without notice to ensure both protocol compliance and data accuracy.

### Data and safety monitoring

Under the guidance of the Department of Defense and COMIRB, an Independent Safety Officer was selected with expertise in acute and critical care to monitor participant safety, evaluate study progress, and suggest changes in design or conduct as needed to address potential safety issues. Adverse events and unanticipated problems will be reported to the Department of Defense Scientific Officer, the Independent Safety Monitor, COMIRB, and the Department of Defense Human Research Protection Office in accordance with their reporting policies. The CCC will provide both the Independent Safety Officer and the Department of Defense Scientific Officer quarterly reports to further monitor trial progress and safety events. No formal interim analyses are required.

### Sample size and power

We estimate an approximate accrual rate of 30 patients per site per month for a total sample size of at least 6000 patients over the 27-month study duration. Based on pilot study data, we estimated a mean SOFD of 15.5 under control conditions with a standard deviation of 11.3 days and an interclass correlation coefficient of 0.04. The estimated sample size of 6000 patients will allow us to detect a difference of 1.42 days in the primary outcome SOFD at 80% power and 1.64 days at 90% power. This assumes that data is normally distributed and that each site contributes the same number of patients as all other sites. As pilot data suggested that some of these assumptions may be false (SOFD is restricted to a specific range, skewed, and bimodal), simulation studies were conducted to address the impact of their violation on power. The simulations, conducted under the less restrictive distributional assumption of SOFD as an ordinal outcome, included removing a fraction of patients during a site crossover period to account for run-in and using the relative trauma patient volume of each site (which varies between 500 and 1500 trauma ICU patients per year) to adjust its sample size relative to the total. These simulation studies demonstrated almost no difference in power estimates relative to traditional power calculations, so power calculations reported above reflect the simpler formula-based approach [20].

## Statistical analysis plan

### Overview of statistical analysis plan (Appendix 3)

We will use descriptive statistics to create summary tables for patient characteristics and outcome variables. We will report the sample size, mean, and standard deviation for all continuous variables stratified by treatment condition (pre- vs post-intervention) and site. Descriptive statistics will exclude missing data. All analysis will be performed on the basis of intention to treat. We define a two-sided threshold for statistical significance of 5%. With a single primary outcome, we will not adjust for multiple comparisons; appropriate caution will therefore be used in interpreting the results of hypothesis testing for secondary analyses.

#### Primary outcome

We will analyze the primary outcome, SOFD, using a linear mixed-effects modeling framework. To account for possible temporal trends associated with intervention implementation at different times, a fixed effect for time will be included. We will account for clustering of patients within sites by including a random intercept term in all models. We will also adjust the model for the following patient-level covariates: age, sex, race and ethnicity, insurance type, Elixhauser Comorbidity Index [21], mechanism of injury, injury severity score, cigarette smoking status, body mass index, and COVID-19 status. We will consider alternative modeling approaches to avoid parametric assumptions while addressing the ordinal nature of these outcomes as needed.

#### Secondary outcomes

Continuous secondary outcomes (i.e., HFD90, VFD28, amount of supplemental oxygen administered, number of hyperoxic/hypoxic episodes) will be analyzed using a linear mixed modeling approach, similarly to the primary outcome. For dichotomous outcomes (i.e., whether or not a patient needed high levels of supplemental oxygen and 90-day in-hospital mortality), we will use a logistic mixed model. For time-to-event outcomes (i.e., time to room air, time to mortality), we will use a Cox proportional hazards regression model with a gamma-distributed random intercept for the site. Time zero will be taken to be the time of arrival in hospital. Kaplan-Meier plots of the time-to-event outcomes will be created to graphically compare distributions between treatment conditions. For ordinal outcomes (i.e., GOS), we will use a mixed-effects ordinal logistic regression model. The proportional odds assumption will be checked to assess if the relationship between the consecutive outcome levels is the same. If violated, a multinomial logit or partially proportional odds mixed-effects model will be used.

#### Missing data

Based on pilot study data, some oxygen exposure and measurements are expected to be missing due to charting inconsistency. In mechanically ventilated patients, we assume that FiO_2_ is maintained constantly until a patient is extubated or a new FiO_2_ is entered. In non-mechanically ventilated patients, we will also assume that the level of supplemental oxygen provided will remain constant until a new value is entered up to 12 h later. However, after 12 h without a new measurement recorded, the patient will be assumed to be on room air. We will not assume an FiO_2_ until the first recorded measurement in the first 12 h. If, after 12 h, a measurement is still not recorded, we will assume the patient is on room air. This will ensure that the primary outcome has no missing data.

### Presentation of outcome data

Table 2 lists the proposed tables and figures for final trial reporting. The results of the trial will be published in peer-reviewed journals.

**Table 2:** Planned figures and tables

Proposed tables (stratified by treatment condition pre- vs post-intervention)	Table 1: Patient characteristics (stratified by treatment
	Table 2: Primary Outcome – SOFD - SOFD (Mean, SD) among survivors - In-hospital mortality (*n*, %) - Alive with 0 SOFD (*n*, %) - Alive with 28 SOFD (*n*, %)
	Table 3: Secondary clinical outcomes - HFD90 - In-hospital mortality to day 90 - VFD28 - Time to room air - GOS - Discharge disposition
	Table 4: Secondary oxygenation outcome - Amount of supplemental oxygen administered - Duration of time on normoxia protocol target - Proportion receiving high levels of supplemental oxygen while in ICU - Duration of time receiving high levels of supplemental oxygen - Duration of time receiving no supplemental oxygen - Incidence of hypoxic and hyperoxic events - Duration of hypoxic and hyperoxic evens
Proposed figures	Figure 1: CONSORT diagram
	Figure 2: Histogram of SOFD stratified by treatment conditions
	Figure 3: Time to mortality stratified by treatment condition (Kaplan-Meier Curve)
Supplemental Tables/Figures	Supplementary Table 1: Patient characteristics by site
	Supplementary Table 2: Primary Outcome – SOFD by site
	Supplementary Table 3: Secondary Outcomes by site
	Supplementary Table 4: Subgroup analysis by trauma subgroup
	Supplementary Table 5: Subgroup analysis by Injury Severity Score

Abbreviations: *GOS* Glasgow Outcome Score, *HFD90* hospital-free days to day 28, *ICU* intensive care unit, *SD* standard deviation, *SOFD* supplemental oxygen-free days, *VFD28* ventilator-free days to day 28

## Discussion

SAVE-O2 is multicenter cluster randomized, stepped wedge implementation trial. This trial will determine if targeted normoxia will safely reduce the need for concentrated oxygen for critically ill trauma patients. The primary outcome will be supplemental oxygen-free days, defined as the number of days alive and not on supplemental oxygen. In addition to answering the primary scientific question, these data will inform military stakeholders on oxygen requirements for critically injured warfighters, while reducing logistical burden in prolonged combat casualty care. This protocol and statistical analysis plan article have been submitted for publication before recruitment was completed.

## Trial status

This article is based on the SAVE-O2 Trauma protocol version 1.1 as of June 3, 2020. Pre-intervention data collection started on July 15, 2020. The first site began the 1-month run-in implementation period on October 15, 2020. The estimated study completion date is December 15, 2022.

## Supplementary Information

**Additional file 1: Appendix 1:** SPIRIT Checklist

**Additional file 2: Appendix 2:** Final Protocol

**Additional file 3: Appendix 3:** Statistical Analysis Plan

## Data Availability

The materials generated during the current study are not publicly available but are available from the corresponding author on reasonable request.

## Abbreviations

CCC: Clinical Coordinating Center
COMIRB: Colorado Multiple Institutional Review Board
DCC: Data Coordinating Center
FiO_2_: Fractional inspired oxygen
GOS: Glasgow Outcome Score
HFD: Hospital-free days
ICU: Intensive care unit
PaO_2_: Partial pressure arterial oxygen
SAVE-O2: Strategy to AVoid Excessive Oxygenation
SOFD: Supplemental oxygen-free days
SpO_2_: Pulse oximetry
VFD: Ventilator-free days
