# Supplementary material for: A multicenter cluster randomized, stepped wedge implementation trial for targeted normoxia in critically ill trauma patients: study protocol and statistical analysis plan for the Strategy to Avoid Excessive Oxygen (SAVE-O2) trial
Source: Trials. 2021 Nov 8;22:784. doi: 10.1186/s13063-021-05688-6 (PMC8574946; doi:10.1186/s13063-021-05688-6)
Supplement: Supplementary file 2 — Additional file 2: Appendix 2: Final Protocol [file 13063_2021_5688_MOESM2_ESM.docx]

COMIRB # 19-2153

Version 1.1

8/14/2020

**Strategy to Avoid Excessive Oxygen (SAVE-O2)**

**for Critically Ill Trauma Patients**

Protocol Version 1.1 (August 14, 2020)

Principal Investigator: Adit Ginde, MD, MPH

Professor, Vice Chair for Research

Department of Emergency Medicine

University of Colorado School of Medicine

Co-Principal Investigator: MAJ Steven G. Schauer, DO, MS

US Army Institute of Surgical Research

Funding: Department of Defense (Joint Warfighter Medical Research Program)

-------------------------------------- --------------------

*SITE Investigator Signature Date*

Table of Contents

[1 ABBREVIATIONS AND DEFINITIONS 4](#_Toc42077421)

[2 TRIAL SUMMARY 5](#_Toc42077422)

[2.1 Title 5](#_Toc42077423)

[2.2 Objective 5](#_Toc42077424)

[2.3 Hypothesis 5](#_Toc42077425)

[2.4 Study Design 5](#_Toc42077426)

[2.5 Inclusion Criteria 5](#_Toc42077427)

[2.6 Exclusion Criteria 6](#_Toc42077428)

[2.7 Endpoints 6](#_Toc42077429)

[2.9 Analysis and Sample Size 6](#_Toc42077430)

[3 BACKGROUND AND RATIONALE 7](#_Toc42077431)

[3.1 Previous Research 7](#_Toc42077432)

[3.2 Preliminary Studies 8](#_Toc42077433)

[4 STUDY OBJECTIVE/HYPOTHESIS 10](#_Toc42077434)

[4.1 Objective 10](#_Toc42077435)

[4.2 Hypothesis 10](#_Toc42077436)

[5 TRIAL DESIGN 10](#_Toc42077437)

[5.1 Cluster Randomized Stepped Wedge Design 10](#_Toc42077438)

[5.2 Run-in Period 11](#_Toc42077439)

[5.3 Participants 11](#_Toc42077440)

[5.4 Recruitment Process and Screening Procedures 11](#_Toc42077441)

[5.5 Informed Consent Process 12](#_Toc42077442)

[5.6 Intervention 12](#_Toc42077443)

[5.7 Timing 13](#_Toc42077444)

[5.8 Implementation 13](#_Toc42077445)

[5.9 Informatics 13](#_Toc42077446)

[6 ENDPOINTS 13](#_Toc42077447)

[6.1 Primary Endpoint 13](#_Toc42077448)

[6.2 Secondary Clinical Endpoints 14](#_Toc42077449)

[6.3 Secondary Oxygenation Endpoints 14](#_Toc42077450)

[6.4 Co-Variates 15](#_Toc42077451)

[7 HUMAN SUBJECTS 15](#_Toc42077452)

[7.1 Waiver of Informed Consent 15](#_Toc42077453)

[7.2 Potential Risks 16](#_Toc42077454)

[7.3 Potential Benefits 16](#_Toc42077455)

[7.4 Data and Safety Monitoring Plan 16](#_Toc42077456)

[8 DATA MANAGEMENT PLAN 17](#_Toc42077457)

[8.1 Clinical Coordinating Center 17](#_Toc42077458)

[8.2 Data Coordinating Center 17](#_Toc42077459)

[8.3 Regulatory Oversight 17](#_Toc42077460)

[8.4 Quality Assurance 17](#_Toc42077461)

[8.4 Data Collection 18](#_Toc42077462)

[8.5 Data Storage 18](#_Toc42077463)

[9 ANALYTIC APPROACH 18](#_Toc42077464)

[9.1 Analysis Plan 18](#_Toc42077465)

[9.2 Missing Data 19](#_Toc42077466)

[9.3 Design Considerations 19](#_Toc42077467)

[Figure 7. Stepped wedge randomization scheme 19](#_Toc42077468)

[9.4 Power 19](#_Toc42077469)

[10 ADVERSE EVENTS 20](#_Toc42077497)

[10.1 Definition of AEs/SAEs 20](#_Toc42077498)

[10.2 Reporting of Adverse Events 21](#_Toc42077499)

[REFERENCES CITED 22](#_Toc42077500)

## 1 ABBREVIATIONS AND DEFINITIONS

**ARDS** = Acute Respiratory Distress Syndrome

**CCATT** = Critical Care Air Transport Team

**CCC =** Clinical Coordinating Center

**CCCRP** = Combat Casualty Care Research Program

**CI** = Confidence Interval

**COMIRB** = Colorado Multiple Institutional Review Board

**CPG** = Clinical Practice Guidelines

**CT** = Computed Tomography

**DCC =** Data Coordinating Center

**DoD** = Department of Defense

**ED** = Emergency Department

**EFIC** = Exception from Informed Consent

**EHR** = Electronic Health Record

**ERC** = En-route care

**FDA** = U.S. Food and Drug Administration

**FiO2** = Fraction of Inspired Oxygen

**GOS =** Glasgow Outcome Score

**HFD90** = Hospital Free Days to Day 90

**HRPO** = Human Research Protection Office

**ICC** = Interclass Correlation Coefficient

**ICU** = Intensive Care Unit

**IRB** = Institutional Review Board

**JTS** = Joint Trauma System

**JWMRP** = Joint Warfighter Medical Research Program

**LAR =** Legal Authorized Representative

**SOFD= Supplemental Oxygen Free Days**

**PaO2** = Partial Pressure of Oxygen in Arterial Blood

**PETAL** = Prevention and Early Treatment of Acute Lung Injury

**PFC** = Prolonged Field Care

**PHI** = Protected Health Information

**PI** = Principal Investigator

**SpO2** = Oxygen saturation via pulse oximetry

**TCCC** = Tactical Combat Casualty Care

**USAMRMC** = US Army Medical Research and Materiel Command

**USSOCOM** = United States Special Operations Command

**VFD28** = Ventilator Free Days to Day 28

## 2 TRIAL SUMMARY

## 2.1 Title

Strategy to Avoid Excessive Oxygen (SAVE-O2) for Critically Ill Trauma Patients

## Objective

The objective is to determine the effectiveness of a multimodal educational intervention to reduce supplemental oxygen use in critically injured patients. We will also evaluate the safety and clinical effectiveness of the more targeted use of oxygen therapy.

# Hypothesis

We hypothesize that a multimodal educational intervention to limit use of excessive supplemental oxygen will reduce exposure to hyperoxia and safely lower the use of concentrated oxygen.

## Study Design

This study will be a multicenter cluster randomized, stepped wedge implementation trial of a multimodal educational intervention to target normoxia in adult trauma patients admitted to the intensive care unit (ICU). Randomization to receive the intervention occurs sequentially at the hospital level for a phased roll-out of the enhanced education and informatics tools to better achieve the consensus-based normoxia target. This well-accepted stepped wedge trial approach is a one-way crossover trial where all sites will ultimately implement the intervention, and the timing of the intervention implementation is randomly ordered. The intervention will be sequentially rolled out to the 8 enrolling sites (i.e., clusters), switching from control to intervention every 3 months at 8 different time points (**Figure 1**).

#### Figure 1. Schematic of the stepped wedge, cluster randomized trial design

We define the consensus-based normoxia target based on thresholds defined in our prior work, which included experts from our proposed sites—oxygen saturation (SpO2) 90-96% and when available, arterial oxygen pressure (PaO2) 60-100 mmHg. The intervention will start in the emergency department upon patient arrival to the hospital and the duration of the intervention period will be the duration of the index ICU stay. The goal is to improve oxygenation to >90% of eligible patient-hours spent in the desired normoxia range, excluding time without supplemental oxygen or time on FiO2 100% and below the normoxia range. Each hospital site will contribute pre-implementation (control) and post-implementation (intervention) data, with the start of the consensus-based intervention period defined by the randomized timing in the stepped wedge design.

# 2.5 Inclusion Criteria

1. Acutely injured patients who meet the criteria for entry into the state or national trauma registry
2. Admission to surgical/trauma ICU within 24 hours of hospital arrival

# 2.6 Exclusion Criteria

1. Age <18 years
2. Prisoners
3. Known pregnancy
4. Transferred patients not admitted through the emergency department

## 2.7 Endpoints

The primary endpoint is supplemental oxygen free days (SOFD) to day 28, defined as number of days alive and not on supplemental oxygen during the index hospitalization, censored at hospital discharge. This outcome has a range of zero days (worst outcome) to 28 days (best outcome). Patients who die during the first 28 days of hospitalization or who are on supplemental oxygen for the first 28 days of hospitalization are assigned zero SOFD. Patients who are discharged without supplemental oxygen or with prior volume of baseline (pre-hospitalization) home oxygen are assumed to remain without additional supplemental oxygen to day 28. Patients who are discharge on supplemental oxygen, if above any prior baseline home oxygen therapy, are assumed to have additional supplemental oxygen to day 28.

Secondary clinical endpoints include: (1) hospital free days to day 90; defined as the number of days alive and outside the hospital between the initial ED visit and 90 days later, censored at hospital discharge; (2) 90 day in-hospital mortality; (3) ventilator-free days to day 28, defined similarly to hospital-free days; (4) time-to-room air, defined as time to achieve no supplemental oxygen; (5) disability at hospital discharge, as measured by the Glasgow Outcome Score with the following five categories: Death, Persistent vegetative state, Severe disability, Moderate disability, Low disability; and (6) discharge disposition.

Secondary oxygenation endpoints will include the amount of supplemental oxygen required during the intervention period, as measured by oxygen supplementation (fraction of inspired oxygen [FiO2] or oxygen volume) over time. We will also measure the proportion of participants and duration of time receiving high levels (>5 liters/minute or FiO2 >40%) of supplemental oxygen and the rate and duration of hypoxic (SpO2 <88%) and hyperoxic (SpO2 >96%).

# 2.9 Analysis and Sample Size

We will analyze the primary endpoint, supplemental oxygen-free days (SOFD) using the generalized linear mixed modeling framework. Continuous secondary outcomes, including hospital-free days, ventilator-free days, oxygen volume required, and number of hypoxic/hyperoxic events will be analyzed similarly. Dichotomous outcomes such as whether a patient requires high flow oxygen (>4 L/min or FiO2 >40%) will be analyzed using a logistic mixed model. The time-to-room air outcome, as well as other time to event analyses (eg, time to mortality) will be analyzed using a Cox proportional hazards regression model with a gamma-distributed random intercept for site. We will analyze the ordinal outcome of GOS using a mixed-effects ordinal logistic regression model. The proportional odds assumption will be checked to assess if the relationship between the consecutive outcome levels is the same, and if violated a multinomial logit mixed-effects model will be used.

The power calculation is based on the primary outcome of supplemental oxygen-free days (SOFD) within 28 days. Preliminary data was used to estimate a mean outcome in the control condition of 15.6; the standard deviation (SD) of the outcome was estimated to be 11.2 and an intraclass correlation coefficient of 0.04. With the full sample of **6000 patients** over the course of the study, we can detect a difference in mean SOFD between control and intervention conditions of 1.41 days at 80% power and a difference of 1.63 days at 90% power. This sample size corresponds to an approximate accrual rate of 25 patients per month per site, assuming 2.5 years of data collection.

# 3 BACKGROUND AND RATIONALE

# 3.1 Previous Research

Oxygen therapy has undisputed importance in the care of critically ill medical and trauma patients to treat and prevent morbidity associated with hypoxia.^1,2^ However, generous supplemental oxygen is routine, and often results in hyperoxia.^3-6^ While there is no known benefit of excessive oxygenation, common clinical perception has been that this practice is safe and creates a margin of safety against hypoxia.^7-9^ Emerging evidence suggests that hyperoxia may be harmful, further accentuating the urgency to determine oxygen titration goals in specific high-risk populations, such as major trauma. Here we summarize the rationale and relevance of the proposed research. Because avoidance of hypoxia is well known and understood in trauma patients, we focus most attention on hyperoxia and the potential need to reduce oxygen exposure.

**Pre-clinical and observational data support the concept of targeted normoxia**. Specifically, there may be a U-shaped relationship between oxygenation and mortality in critically ill patients, with both ends of the spectrum—hypoxia and hyperoxia—independently associated with higher mortality.^10,11^ While the rationale to avoid hypoxia is clear, the risks of hyperoxia may be less obvious. Laboratory evidence has long demonstrated toxicity associated with hyperoxia through mitochondrial production of pro- inflammatory reactive oxygen species and ischemic vasoconstriction that leads to tissue damage and vital organ injury.^12-14^

**Numerous observational cohort studies** have demonstrated an association between hyperoxia and higher mortality broadly in intensive care unit patients and in specific subpopulations, such as cardiac arrest, myocardial infarction, ischemic stroke, and traumatic brain injury.2,15 Prehospital hyperoxia can also have an important impact on clinical outcomes.16 In a recent meta-analysis, hyperoxia (compared to normoxia) was associated with a 21% higher odds of mortality across a broad range of critical illness, even after adjusting for baseline characteristics and illness severity.15

However, beyond traumatic brain injury (for which both hypoxia17 and hyperoxia15 are associated with higher mortality), there is little evidence for the relationship between oxygenation and outcomes in major trauma, and therefore clinicians currently leverage evidence from other conditions and expert consensus.

**Preliminary clinical trial evidence in non-traumatic conditions supports the safety and potential efficacy of targeted normoxia**. Panwar et al. recently published the first randomized controlled trial to compare different oxygen targets in critically ill patients, comparing a target oxygen saturation (SpO2) 88- 92% (“conservative”) vs. ≥96% (“liberal”/conventional).^18^ In this multicenter pilot trial (4 intensive care units [ICU] in Australia/New Zealand; n=103), targeted normoxia (conservative group) safely reduced the amount of supplemental oxygen administered (mean FiO2 >10% lower in the conservative group during the first 7 days) and duration of time in the hyperoxic range (4% conservative vs 22% liberal) without an increase in time spent with SpO2 <88% (0.3% conservative vs 1% liberal). In this small, underpowered trial, clinical outcomes (organ dysfunction, hospital length of stay, mortality) were not statistically different between groups. However, there were promising signals for targeted normoxia reducing 90-day mortality (adjusted hazard ratio 0.77 [95%CI, 0.40-1.50]), especially in the pre-specified subgroup with acute lung injury (adjusted hazard ratio 0.49 [95%CI, 0.20-1.17]).

Girardis et al. similarly compared a targeted normoxia strategy (arterial oxygen pressure [PaO2] 70-100 mmHg or SpO2 94-98%) to a conventional (relative hyperoxic) strategy (PaO2 ≥150 mmHg or SpO2 ≥97%) in a single Italian ICU (n=434).^10^ The targeted normoxia group had substantially lower ICU mortality (11.6% vs. 20.2% in the conventional group; p=0.02) and a lower incidence of hospital mortality (24.2% vs 33.9%; p=0.03), shock (3.7% vs. 10.6%; p=0.006), and duration of mechanical ventilation (mechanical ventilation free hours 72 vs. 48; p=0.02).

Most recently, the ICU-ROX investigators conducted a randomized trial of conservative oxygen therapy (focusing on avoidance of SpO2 ≥97%) compared to usual unstructured oxygen therapy in 1000 mechanically ventilated patients from 21 ICUs in Australia and New Zealand.^19^ The conservative oxygen therapy group had substantively lower supplemental oxygen administration, and no difference in mortality or ventilator free days between the two groups, although there was a suggestion of benefit in patients with hypoxic-ischemic encephalopathy. This study also did not focus on trauma patients and enrolled mostly post-operative and acute brain disease patients.

Additional clinical trial evidence in other populations suggests that targeting either hypoxia or hyperoxia may be harmful. In three high-profile randomized trials of pre-term infants, permissive hypoxia (SpO2 85-89%) was associated with a higher mortality and disability.^20-22^ However, these trials are unlikely applicable to critically injured adults; in addition, we propose a normoxia, rather than permissive hypoxia, strategy Similarly, five clinical trials have demonstrated worse clinical outcomes of hyperoxia in non-critical trauma,^23^ myocardial infarction,^24^ abdominal surgery,^25^ septic shock (NCT01722422), and ischemic stroke (NCT00414726) patients. Therefore, the prehospital, emergency, and critical care communities now recommend careful titration to normoxia to avoid hypoxia and preserve tissue oxygenation while preventing iatrogenic hyperoxia.^26,27^ Collectively, these trials provide strong rationale for the feasibility, safety, and potential efficacy for a targeted normoxia strategy in critically ill trauma patients.

**Implementation of targeted normoxia is desirable, safe, and feasible, and serves as a platform to study oxygenation targets in critically ill trauma patients**. Prehospital, emergency, and critical care physicians now firmly believe that avoidance of hypoxia and hyperoxia are both important and that oxygen titration should be practiced in critically ill patients.^28,29^ Several implementation studies demonstrate that oxygen titration based on non-invasive oxygen saturation can be safely protocolized to achieve normoxia, ^26,27,30^ and markedly reduce consumption of supplementary oxygen.^4,19^. Accordingly, targeted normoxia is now widely accepted as standard care in emergency departments and ICUs.^12-14^ For example, ARDS Network oxygenation targets (SpO2 88-95%) has been widely accepted for patients with and without ARDS.^31^ Yet, without specific protocols, widespread use of excessive oxygenation persists in routine care in the prehospital setting, emergency departments, and ICUs with over 75% of patients exposed to prolonged periods of hyperoxia.^12,32^ The goal of the current study is to implement a multimodal educational intervention to limit use of excessive supplemental oxygen and reduce exposure to potentially harmful hyperoxia.

# Preliminary Studies

***Our prior USSOCOM-funded work in this area*** provides the foundation for the current proposal. First, we published a **systematic review** of 43 studies (17 trauma, 26 non-trauma critical illness) relevant to oxygenation and outcomes in critically injured patients.38 Our results support an overall association between both hypoxia and hyperoxia with worse clinical outcomes, suggesting that a normoxia approach (avoiding both hypoxia and hyperoxia) is likely optimal. However, our systematic review most importantly highlighted the lack of trauma-specific data to determine ideal oxygen targets. Second, we led a **Delphi consensus process** of 31 nationally and internationally recognized military and civilian experts in trauma surgery, emergency medicine, critical care, and military operational medicine (manuscript in preparation).


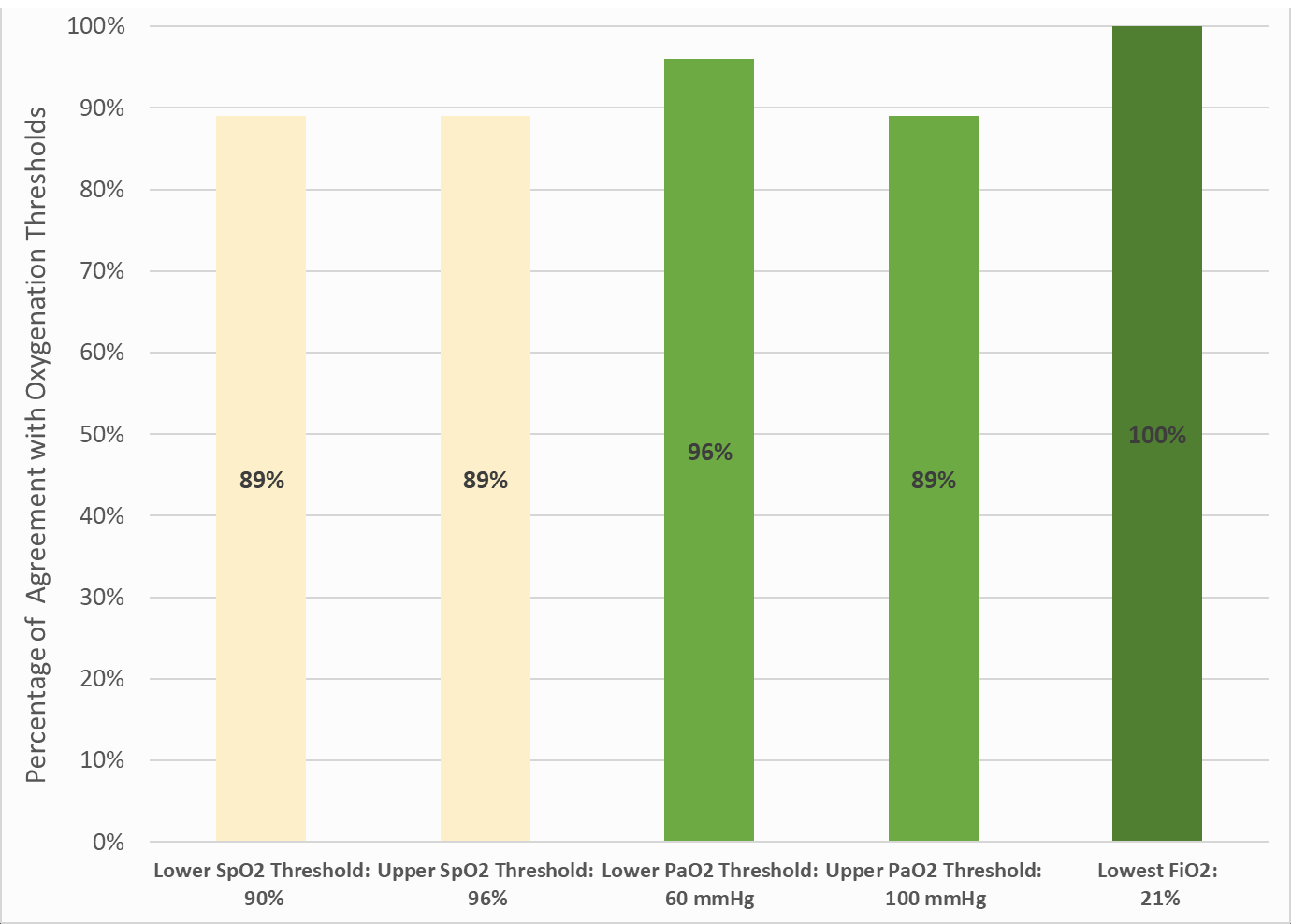


**Figure 2. Final Vote to Establish Consensus on SpO2, PaO2, and Lowest Acceptable FiO2 Thresholds**

We asked the expert panel to rate how strongly they agreed or disagreed with specific SpO2 low thresholds, SpO2 high thresholds, PaO2 low thresholds and PaO2 high thresholds. Based on our analysis of the data, we were able to identify what the majority of experts felt were appropriate oxygenation thresholds. After narrowing the proposed SpO2 and PaO2 ranges, as well as the lowest acceptable FiO2 to maintain these ranges, we conducted a final vote of the expert panel, as summarized in **Figure 2**. Our findings demonstrated nearly unanimous support for a targeted normoxia strategy for oxygenation of critically injured patients and developed a consensus-based definition of normoxia, based on a goal oxygen saturation of 90-96% and PaO2 of 60-100 mmHg. We will evaluate the impact of the educational intervention to improve achievement of this target.

Third, we conducted a **retrospective observational cohort** study of critically ill trauma patients in three Colorado trauma centers to determine the incidence of modifiable hyperoxia and its association with outcomes. We found that most critically ill trauma patients in the emergency department and ICU receive supplemental oxygen and are hyperoxic (**Figure 3**).

**Figure 3. Frequency of hyperoxia (SpO2 >96%) and oxygen use in critically ill trauma patients**


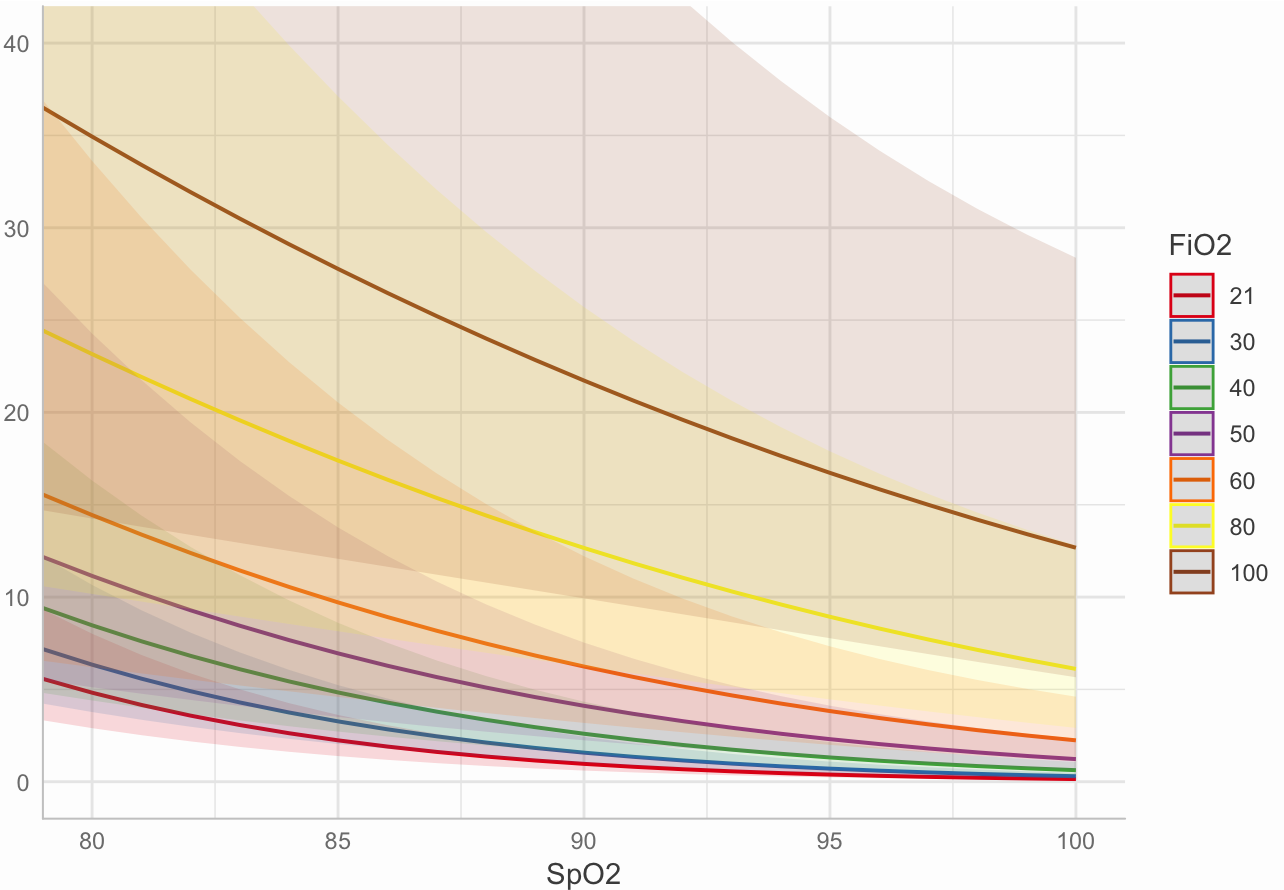
In addition, higher oxygenation supplementation (FiO2) was associated with increased mortality at all SpO2 levels, after adjusting for injury severity and other covariates (**Figure 4**).

Adjusted Mortality Risk

**Figure 4. Association between oxygen supplementation, oxygen saturation, and adjusted mortality risk**

Finally, we evaluated a **pilot quality improvement intervention,** at the University of Colorado Hospital Level 1 Trauma Center supporting the feasibility and safety of the targeted normoxia approach (NCT03789396). The protocol (which will also be leveraged in the proposed research) was approved by our local IRB (COMIRB #18-1528) and the DoD Human Research Protection Office (HRPO, Log Numbers A-20617.3a and A-20617.3b). This trial was deemed minimal risk and approved under a waiver of informed consent. After implementation, we found a significant reduction in oxygen consumption without any evidence of increase in hypoxia events (**Figure 5**). We will leverage this experience to test a multimodal educational intervention to reduce exposure to excessive supplementation oxygen and avoid hyperoxia.


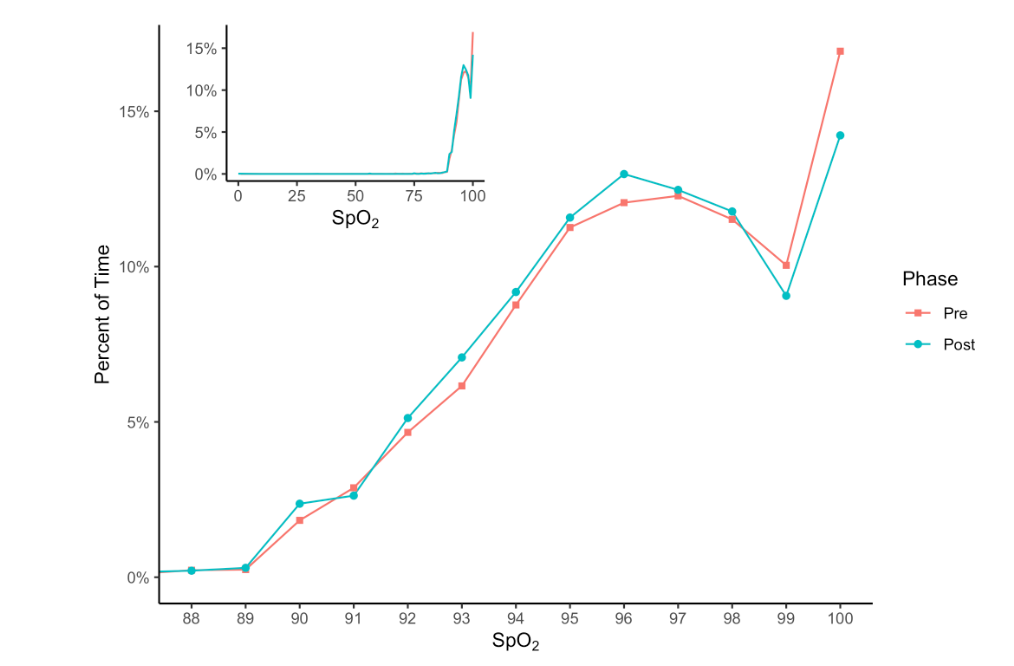


**Figure 5. Reduced exposure to hyperoxia after pilot quality improvement intervention**

# 4 STUDY OBJECTIVE/HYPOTHESIS

## 4.1 Objective

The objective is to determine the effectiveness of a multimodal educational intervention to reduce supplemental oxygen use in critically injured patients. We will also evaluate the safety and clinical effectiveness of the more targeted use of oxygen therapy.

## 4.2 Hypothesis

We hypothesize that a multimodal educational intervention to limit use of excessive supplemental oxygen will reduce exposure to hyperoxia and safely lower the use of concentrated oxygen.

# 5 TRIAL DESIGN

## 5.1 Cluster Randomized Stepped Wedge Design

This study will be a multicenter cluster randomized, stepped wedge implementation trial of a multimodal educational intervention to better achieve the consensus-based normoxia target in adult trauma patients admitted to the intensive care unit (ICU). Randomization, defined as crossover from pre-intervention usual care to the intervention-enhanced usual care, occurs at the hospital-level for a phased roll-out of the targeted normoxia intervention to each hospital site sequentially.

Accordingly, this well-accepted stepped wedge trial approach is a one-way crossover trial where all sites will ultimately implement the educational intervention, and the timing of the intervention implementation is randomly ordered. The intervention will be sequentially rolled out to the 8 enrolling sites (i.e., clusters), switching from control to intervention every 3 months at 8 different time points (**Figure 6**).

**Figure 6. Schematic of the stepped wedge, cluster randomized trial design**

## 5.2 Run-in Period

The design incorporates a 1-month transition period for staff education/implementation at each hospital, during which training will be delivered and to allow for full implementation of the education and consensus-based intervention. The CCC and overall PIs will provide standardized education and materials to local research teams for site implementation. During the transition period, the cluster cannot be considered as either receiving the structured usual care intervention or the unstructured usual care control, and thus patients treated in the emergency department and ICU during that period will not be enrolled or included in the analysis.

## 5.3 Participants

Hospitals eligible for participation have endorsed the consensus-based normoxia recommendation of SpO2 90-96% for critically ill trauma patients but currently have no specific plans or resources to promote this oxygenation target during the phased implementation of the enhanced educational intervention. We will define the target population as acutely injured patients who meet criteria for entry into the state/national trauma registry and are admitted to the surgical/trauma ICU within 24 hours of hospital arrival. We anticipate that patients will present primarily to the participating trauma centers emergency departments, although we will include patients transferred to the participating emergency department from another hospital. We will exclude transferred patients who are not admitted through the emergency department. Additional exclusion criteria are age <18 years, prisoners, or known pregnancy (we expect that all major burn female patients of childbearing age will receive a pregnancy test per usual care as part of standard protocols). We will include both mechanically ventilated and non-mechanically ventilated patients. We will pre-define specific trauma subgroups, including hemorrhagic shock, mechanical ventilation, injury severity/mechanism, and traumatic brain injury.

## 5.4 Recruitment Process and Screening Procedures

Implementation of the educational intervention to improve adherence to the consensus-based normoxia target occurs at the site-level, is administered by local clinical/operations team, and is facilitated by the local research team. The intervention is site-level implementation of the educational intervention and is not conducted on an individual patient level. Therefore, the research will be performed under a waiver of informed consent for minimal risk research (see Section 7.1). The local research team will facilitate education, training, and monitoring by clinical operations leadership to implement the intervention, but individual patients will not be actively screened and enrolled prospectively. Treatment decisions are informed by the consensus-based recommendations but are not binding and completely at the discretion of the treatment clinicians in the best interest of the patient. Each site will work with the local informatics team and trauma registrars to identify subjects that qualify for inclusion into the retrospective data collection and analysis.

## 5.5 Informed Consent Process

This research meets the federal definition and ethical standards for waiver of informed consent. Specifically, according the Common Rule (45 CFR 46) that governs the ethical conduct and oversight of human subjects research, section 116(d) indicates that a waiver of informed consent can be obtained if the research involves no more than minimal risk to subjects, waiver of consent will not adversely affect the rights and welfare of subjects, and the research could not practicably be carried out without the waiver of consent. This approach is commonly accepted in implementation science studies,^36,37^ such as the current proposal. In addition, recommendations are not binding and decisions about oxygenation for individual patients are at the discretion of the treating clinicians in the best interests of the patient. We will monitor and record the reasons for alternate oxygenation strategies

DODI 3216.02 requires that, when altering the informed consent process, all participants must derive benefit from implementation into this study. To this end, our intervention is occurring at the institutional level, and we are seeking to implement what we believe represents the consensus-based optimal care with regards to oxygen supplementation. After the intervention, *all participants*, will receive the intervention that is supported by a systematic review, a consensus panel of experts, and a previous pilot study. Thus, by virtue of institution-wide implementation of a protocol for optimizing oxygen supplementation all participants will derive benefit. The pre-intervention represents care that has already occurred, and subject “participation” will not occur until after their care is complete – this represents retrospective data collection.

## 5.6 Intervention

We will implement the multimodal educational intervention to better achieve targeted normoxia based on thresholds defined by our expert consensus panel in our prior work —SpO2 90-96% and PaO2 60-100 mmHg (when available). Each hospital site will contribute pre-implementation (control) and post-implementation (intervention) data, with the start of the intervention period defined by the randomized timing in the stepped wedge design. Based on our prior work, while expert consensus and local operational leaders strongly recommends normoxia, current practice often employs excessive oxygen supplementation to the hyperoxia range. The primary reason for this is convenience and dogma, as key opinion leaders and expert consensus strongly suggest that targeting normoxia is optimal for patient care. Thus, the intervention focuses on implementing educational and informatics tools to better achieve the desired normoxia target.

The intervention is a multimodal approach, tailored to local site preferences, using a combination of education and training of the clinical teams via staff meetings, floor rounds, morning and evening huddles, flyers for staff areas, and educational videos. The research team will educate sites on the development of electronic alerts/reminders, according to site preferences. We will provide sites with audit-feedback reports to educate the staff of their progress towards titration of oxygen supplementation to the defined normoxia range for patients in the post-implementation phase, while also identifying areas in need of improvement and re-education needs to move towards the defined normoxia range. We do not anticipate that all patients will be oxygenated in the target range and thus will promote a goal of at least 90% of patient-hours within the normoxia target, excluding time already on no supplemental oxygen but above the target range or at 100% FiO2 but below the target range (as these are non-modifiable).

All sites begin in the control phase, where they receive unstructured usual care (typically higher oxygen supplementation and more frequent hyperoxia). When sites reach their randomly assigned time to transition to the intervention, there will be a one-month run-in education and training period, which does not contribute to data collection, after which the site will remain in the intervention phase for the duration of the trial.

The treating clinical team (primarily nurses and respiratory therapists) will implement the intervention through oxygen titration for individual patients. Interventions for treatment of hypoxia will follow usual local practice. Interventions for treatment of hyperoxia (SpO2 >96% or PaO2 >100 mmHg) will involve down titration of FiO2 (or supplemental oxygen for non-mechanically ventilated patients) within a time frame based on local site preferences—typically in increments of no greater than 0.10 until goal oxygenation in the normoxia range is achieved or the patient is on FiO2 0.21 (including room air [no supplemental oxygen] for non-mechanically ventilated patients).

## 5.7 Timing

The intervention will start in the emergency department upon patient arrival to the hospital, and the duration will last the entire ICU stay. This timing focus efforts on active oxygen titration early in the post-injury course for critically ill trauma patients when therapeutic momentum is established. The goal adherence to the protocol is >90% of eligible patient-hours spent in the normoxia range, excluding time without supplemental oxygen (FiO2 0.21 or on room air) but above the target range or time on FiO2 100% but below the target range (as these are non-modifiable).

## 5.8 Implementation

We will leverage the implementation educational materials and protocols developed in our pilot trial to titrate oxygen supplementation to the target normoxia. The unit level consensus- based best practice intervention will be administered primarily by respiratory therapists and nurses, following protocolized electronic clinical decision support and automated feedback. The intervention is not binding, and clinical decisions can always override the recommendations when it is in the best interest of the patient (e.g., non-decompressed pneumothorax, carbon monoxide poisoning). The implementation at each site will involve physician, advanced practice providers, nursing, and respiratory therapy champions and engagement from leadership in the emergency department, trauma surgery, and surgical/trauma ICU to emphasize and prioritize the intervention. Because hypoxia is generally well addressed, the focus of the intervention will be down-titration of oxygen when patients are in the hyperoxia range to avoid excessive oxygen supplementation.

## 5.9 Informatics

The implementation will typically involve a new build for a ‘best practice alert’ in the electronic medical record that identifies eligible participants with oxygenation measurements (SpO_2_ or PaO_2_) outside the target normoxia range (both hypoxia and hyperoxia). Oxygen titration will be based on standard clinical practice at the local site, including FiO_2_ and positive end expiratory pressure for mechanically ventilated patients and oxygen flow rate for patients that are not mechanically ventilated (unless no additional oxygen titration can be made). These reminders will alert respiratory therapist and nurses to titration oxygenation to the target range. Alerts can be disabled for specific patients due to clinical circumstances requiring oxygenation outside the target range. In addition, weekly electronic reports at the site level will document oxygenation within and outside of the target normoxia range, compared to prior performance at the site and contemporaneous performance at other participating sites.

## 6 ENDPOINTS

In both the pre- and post-implementation phases, we will follow patients for a period of 90 days or until hospital discharge to determine the effect of the interventions on important primary and secondary clinical outcomes. Both pre-implementation and post-implementation data will be collected retrospectively from all sites to ensure that prospective implementation does not bias the final data collection. Thus, the start of the study will be the same at all sites, though the timing of implementation of the intervention will vary based on the randomization. The individuals enrolled at the end of the last phase of the study will continue to be followed for 90 days to obtain follow-up on their clinical outcomes.

# Primary Endpoint

The primary endpoint is **supplemental oxygen free days (SOFD)** to day 28, defined as number of days alive and not on supplemental oxygen during the index hospitalization, censored at hospital discharge. This outcome has a range of zero days (worst outcome) to 28 days (best outcome). Patients who die during the first 28 days of hospitalization or who are on supplemental oxygen for the first 28 days of hospitalization are assigned zero SOFD. Patients who are discharged without supplemental oxygen or with prior volume of baseline (pre-hospitalization) home oxygen are assumed to remain without additional supplemental oxygen to day 28. Patients who are discharge on supplemental oxygen, if above any prior baseline home oxygen therapy, are assumed to have additional supplemental oxygen to day 28.

# 6.2 Secondary Clinical Endpoints

All secondary outcomes are assessed until 90 days after initial hospital presentation or at hospital discharge, whichever occurs first.

1. **Hospital-free days to day 90 (HFD90)**, defined as the number of days alive and outside the hospital between the initial ED visit and 90 days later, censored at hospital discharge. This outcome is a composite of in-hospital mortality and length of hospital stay and has a range of 0 days (worst outcome) to 90 days (best outcome). Patients who die in the hospital within the 90-day observation period or are in the hospital for the entire study are assigned 0 hospital-free days. Patients discharged prior to day 90 are assumed to survive to day 90 and are assigned [90 – hospital length of stay] hospital-free days.
2. **In-hospital mortality to day 90**, defined as a dichotomous vital status (survived or died) at hospital discharge or day 90, whichever is first
3. **Time to mortality to day 90**, based on vital status and date of death and censored at hospital discharge or day 90, whichever is first
4. **Ventilator Free Days to day 28 (VFD28)**: VFD depends on both duration of ventilation and mortality through study day 28. In participants who survive 28 days, VFD is defined as 28 minus duration of ventilation. Duration of ventilation is counted from the first study day of invasive mechanical ventilation (IMV) through the last day of IMV provided the last day is prior to day 28. Otherwise, it is counted from the first study day of IMV through day 28. For participants discharged with IMV (e.g., to LTAC facility) prior to day 28, the patient will be assumed to require IMV through day 28 (zero VFD will be assigned). Participants discharged from the hospital prior to day 28 (but not to home) on unassisted breathing will be assumed to remain on unassisted breathing through day 28. Isolated periods of ventilation briefer than 24 hours for surgical procedures and ventilation solely for sleep disordered breathing do not count towards duration of ventilation. In participants who never require assisted breathing, duration of ventilation is zero (28 VFD assigned). Participants who do not survive 28 days will be assigned zero VFD.
5. **Time to Room Air**, defined as the time from hospital presentation to the first episode of no supplemental oxygen (FiO2 0.21 or room air).
6. **Glasgow Outcome Score** **(GOS),** as assessed by chart review at hospital discharge with one of the following five categories: Death, Persistent vegetative state, Severe disability, Moderate disability, Low disability
7. **Discharge Disposition**, defined as home (return to prior level of care) or facility (e.g, acute rehab, skilled nursing facility)

## 6.3 Secondary Oxygenation Endpoints

1. **Amount of supplemental oxygen administered**, defined as total estimated oxygen volume while in the ICU after hospital arrival
2. **Duration of time on normoxia protocol target**, defined as SpO2 90-96% or receiving no supplemental oxygen ((FiO2 0.21 or room air) while in the ICU
3. **Proportion of participants receiving high levels of supplemental oxygen** (FiO2 >0.40 or >4 liters per minute) for >2 hours while in the ICU This excludes time in the operating room.
4. **Duration of time receiving high levels of supplemental oxygen** (FiO2 >0.40 or >4 liters per minute) **while in the ICU**
5. **Duration of time receiving no supplemental oxygen** (FiO2 0.21 or room air) while in the ICU
6. **Incidence of hypoxic events** (SpO2 <88%) while in the ICU
7. **Duration of hypoxic events** (SpO2 <88%) while in the ICU
8. **Incidence of hyperoxic events** (SpO2 >96%) while in the ICU
9. **Duration of hyperoxic events** (SpO2 >96%) while in the ICU

## 6.4 Co-Variates

To adjust for baseline characteristics and severity of illness, we will collect demographics (age, sex, race/ethnicity, payer), military status, mechanism of injury, injury severity score, shock index, Elixhauser comorbidity index, cigarette smoking status, body mass index, and COVID-19 status. We will use standard, validated methods to extract these data.

## 7 HUMAN SUBJECTS

The Colorado Multiple Institutional Review Board (COMIRB) will serve as the central IRB for all implementation sites. We will create reliance agreements with IRBs for the eight enrolling sites to cede review to COMIRB.

## Waiver of Informed Consent

This research meets the federal definition and ethical standards for waiver of informed consent. Specifically, according to the Common Rule (45 CFR 46) that governs the ethical conduct and oversight of human subject’s research, section 116(d) indicates that a waiver of informed consent can be obtained if the research involves:

1. No more than minimal risk to subjects

We are implementing an educational intervention to better achieve a consensus-based optimal oxygenation target, based on a systematic review, Delphi consensus process, pilot study, and additional outreach to enrolling sites. Hospitals eligible for participation have endorsed the consensus-based normoxia recommendation of SpO2 90-96% for critically ill trauma patients but currently have no specific plans or resources to promote this oxygenation target during the phased implementation of the enhanced educational intervention. The intervention is site-level implementation of the educational and informatics tools and is not conducted on an individual patient level. Treatment decisions about oxygenation for individual patients are informed by the consensus-based recommendations but are not binding and completely at the discretion of the treatment clinicians in the best interest of the patient.

Prior to implementation of the intervention, the care delivered would be the same as that delivered in the absence of the research study, and data collection is retrospective. The intervention will facilitate implementation of the educational and informatics tools using resources that would not be available to sites in the absence of the research study. In addition, it takes approximately 3 months to fully implement the intervention at each site (facilitated by the Clinical Coordinating Center at University of Colorado Denver), thus simultaneous implementation of the intervention is not feasible and as above, usual care is provided in the pre-implementation phase at all sites.

Therefore, in both the pre-intervention and post-intervention phases, the research itself introduces no more than minimal incremental risk beyond the condition and usual care in the absence of research, as defined by the Common Rule definition of minimal risk research.

1. Waiver of consent will not adversely affect the rights and welfare of subjects

All patient care, including oxygenation, will occur at the discretion of the treating clinical team. The educational intervention seeks to better inform clinicians to reduce excessive oxygen supplementation and minimize exposure to hyperoxia that occurs during current usual care. In these circumstances, it is permissible to alter informed consent with provision of information to subjects or surrogate decision-makers, when feasible. We will follow a typical approach of ‘broadcast notification’ of the institution’s involvement in the research focused on oxygen titration through signage in the participating units (see protocol attachment). Contact information for the local research team is provided for questions.

In addition, individual clinicians are not being studied and therefore not considered research subjects in this study. Data collection is aggregated at the unit-level, not at the provider-level.

1. The research could not practicably be carried out without the waiver of consent.

The educational intervention is provided at a hospital unit-level, not at the patient-level. The subjects receive usual care in the pre-implementation phase and education-enhanced usual care in the post-intervention phase. The research team has no interaction with the patients or their families and therefore, obtaining written consent would not be feasible, particularly given the large number of patients who receive care in these units. In addition, we focus on patients admitted to the ICU, representing a critically ill patient population, many of whom will be intubated or not able to make decisions very early in their clinical care. Optimizing their oxygenation starts in the ED where it will not be practical to consent many patients due to their mental status or the urgent need for intervention and identifying legally authorized representatives for clinical care or research in critically ill trauma patients is often not possible.

## 7.2 Potential Risks

The educational intervention focuses on better achieving consensus-based optimal oxygenation targets and particularly avoiding exposure to potentially harmful hyperoxia. There may be a small risk of increased exposure to hypoxia episodes, but we did not observe this in our pilot study and this will be closely monitored. The research team will help to set up alerts and reporting to help the treating clinicians minimize the risk of exposure to both hypoxia and hyperoxia. There is also a risk for loss of confidentiality, including inappropriate disclosure of identifiable health information. However, we have taken steps to prevent this occurrence, including use of REDCap for data management, only providing access to investigators and research staff directly involved in the project, providing access to identifiers only at the local site level, and removing identifiers from the dataset at the earliest possible opportunity. If data needs to be transferred outside of REDCap, we will used institutionally approved methods for file transfer and de-identify data prior to transfer.

## 7.3 Potential Benefits

The educational intervention has the potential to benefit individual patients by reducing potential harms associated with both hyperoxia and hypoxia. The results will help to provide additional data on the impact of reducing supplemental oxygen use and reducing exposure to hyperoxia. Overall, this research has potential to improve the care of civilian and military trauma patients, as well as to improve logistics/combat missions for the Department of Defense (DoD).

# 7.4 Data and Safety Monitoring Plan

This data safety and monitoring plan is commensurate with the risks as well as the size and complexity of the study. With input and approval from the DoD and COMIRB, we will select an Independent Safety Officer with expertise in acute and critical approval of the DoD and COMIRB to act in an advisory capacity to monitor participant safety, evaluate the progress of the study, and suggest changes to the study design or conduct to abrogate any safety issues.

This plan outlines the appropriate oversight and monitoring of the conduct and progress of the study to ensure that important information that may affect the safety and welfare of subjects is collected, recognized, and acted upon quickly while still ensuring the validity and integrity of the data. This monitoring plan includes structured adverse event (AE) and serious adverse event (SAE) determination, monitoring and reporting. AEs and unanticipated problems (UPs) will be reported to the DoD Scientific Officer, the Independent Safety Monitor, COMIRB, and the DoD HRPO, in accordance with their reporting policies. The Principal Investigator (PI) globally and the site PI at the local level will be responsible for ensuring participants’ safety on a daily basis.

The Independent Safety Officer will review unexpected SAEs or other major unanticipated or safety-related issues within 3 business days of receipt and have the ability to request additional information as needed. The CCC will provide the Independent Safety Officer and the DoD Scientific Officer a quarterly report beginning with protocol implementation at the first site to further monitor trial progress and safety events. In addition, the CCC will provide an annual report with cumulative enrollment and safety data to the Independent Safety Officer, DoD Scientific Officer, COMIRB and the DoD HRPO at the time of continuing review. Because the focus of the intervention is on achieving oxygenation targets (particularly reducing exposure to hyperoxia), and summary implementation and clinical data will be provided quarterly, we do not believe that formal interim analyses is required.

## 8 DATA MANAGEMENT PLAN

## 8.1 Clinical Coordinating Center

The Clinical Coordinating Center (CCC) at the University of Colorado Denver, led by Dr. Ginde (overall Principal Investigator), will oversee all aspects of the trial and site activities. Specifically, the CCC will lead 60-minute biweekly meetings with the entire team of site investigators and coordinators to review progress and timelines, using structured statement of work chart to ensure optimal roll-out of site randomization and to review recruitment goals and metrics. The CCC will coordinate directly with eight selected sites to launch data collection and randomization and develop/implement standardized training procedures, in conjunction with site investigators and coordinators

## Data Coordinating Center

The Data Coordinating Center (DCC) at Vanderbilt University Medical Center, led by Dr. Chris Lindsell and Dr. Paul Harris, will provide specialized REDCap-related data planning, implementation, and management services to the CCC and trial implementation sites. For this project, the DCC will focus specifically on REDCap database development, socialization and implementation of digital forms, data mapping and sharing of maps between electronic health record (EHR) and REDCap environments, hosting digital training sessions for the REDCap-FHIR module; creating in-depth documentation for installation and manipulation as required for the study; and maintaining ample correspondence with involved parties for questions and concerns throughout the study’s lifespan. The DCC will work closely with the CCC biostatistical core in developing on-study data reports and the data analysis plan.

## Regulatory Oversight

The Colorado Multiple Institutional Review Board (COMIRB) will serve as the Central IRB (cIRB) for the trial. All performance sites will cede to the cIRB. The CCC will be responsible for dissemination of study protocols and procedures and providing training materials for all investigators and clinicians involved in the trial. We will use both vertical and horizontal training approaches, where CCC research coordinators will be directly involved in training research staff at the eight clinical sites. Site research staff will then be responsible for training their clinical and additional research staff, although CCC research coordinators will provide close oversight and monitoring of the training of staff for all sites. Site training will include Good Clinical Practice

(GCP) using standardized modules from the Collaborative Institutional Training Initiative (CITI) Program, which is designed to prepare research staff in the conduct of clinical trials with human participants. All participating staff will be required to complete specified trainings prior to enrollment of patients. The required training will occur at the direction of their local regulatory office.

## Quality Assurance

The CCC will perform random spot checks of patient records for enrollment considerations, protocol compliance, and data accuracy. However, these audits will be infrequent and limited in scope by necessity. Therefore, to increase the frequency and expand the breath of protocol compliance review, sites will also implement their own internal audit process. After an internal audit at a site is conducted, the local research team along with CCC will identify areas of improvement when necessary.

## 8.4 Data Collection

The source of data collection will primarily be the electronic health record and the state trauma registry data at the local implementation site. Informatics experts at the local site will facilitate mapping variables, in collaboration with local site experts and the DCC. Manual data extraction will not be required, however manual verification of electronically captured data will enhance data fidelity. The CCC will train local sites on data collection to ensure consistency and monitor data quality. We will collect all available oxygen data, including all SpO2, PaO2, FiO2, PEEP, and oxygen volume measurements recorded in the electronic health record during the hospital stay. In addition, at capable sites, we will collect raw device-validated data for continuous SpO2 measurements using internal algorithms that validate the plethysmograph (waveform). Because there is some inherent bias to data that is ultimately recorded by humans in the electronic health record, device data will help us to perform additional sensitivity analyses to ensure robust and valid results. Other demographic, clinical, and injury related data to describe baseline characteristics and outcomes should be readily available from the local EHR and trauma registry.

## 8.5 Data Storage

We will collect, manage, and store human subject’s data using a REDCap database that the CCC and DCC will develop for this study**.** REDCap is a secure and encrypted web application designed to support data capture for research studies, providing user-friendly, web-based case report forms, real-time data entry validation (e.g. for data types and range checks), audit trails and a de-identified data export mechanism to common statistical packages (SAS, Stata, R/S-Plus). The system was developed by a multi-institutional consortium which includes University of Colorado Denver and was initiated at Vanderbilt University. The database will be hosted at the Vanderbilt University DCC and shared with the University of Colorado Denver CCC for on-study reporting and data analysis. Protected Health Information (PHI) will be accessible only to local sites and not shared with the DCC or CCC.

## ANALYTIC APPROACH

As an overall approach, we will analyze primary and secondary endpoints using a mixed effects modeling framework, with specific distributions chosen depending on the type of outcome (e.g., binary, count, ordinal, time-to-event). The primary analysis will be the effect of the treatment condition on supplemental oxygen-free days. With the stepped-wedge design of the study, the intervention is implemented at different times at the different hospitals; therefore, a fixed effect for time will be included to adjust for possible temporal trends. We will account for clustering of patients within sites by including a random intercept term in all models. To improve precision, we will adjust final models for pre-selected patient-level covariates (e.g., age, gender, race/ethnicity, payer, Elixhauser Comorbidity Index, mechanism of injury, Injury Severity Score, cigarette smoking status, body mass index, COVID-19 status). Subgroup analyses will be conducted by including interactions between the treatment condition and the specific covariate of interest (e.g., trauma subgroups, categories of injury severity score). For all hypotheses, unless explicitly stated otherwise, significance tests will be two-sided at the 5% significance level. There is a single primary endpoint, and we will not adjust for multiple comparisons. Analyses will be conducted in SAS or R.

## Analysis Plan

We will analyze the primary endpoint, supplemental oxygen-free days (SOFD) using the generalized linear mixed modeling framework. We will begin by assuming a normal distribution for the outcome, but will assess if other distributions, such as negative binomial or log-gamma, provide a better fit. If there is a larger proportion of patients who experience mortality than expected, zero-inflated mixed models may be considered. We will also consider alternative modeling approaches such as cumulative logit mixed models treating the response as an ordinal outcome to avoid more parametric assumptions.

Continuous secondary outcomes, including hospital-free days, ventilator-free days, oxygen volume required, and number of hypoxic/hyperoxic events will be analyzed similarly. Dichotomous outcomes such as whether a patient requires high flow oxygen (>4 L/min or FiO2 >40%) will be analyzed using a logistic mixed model. The time-to-room air outcome, as well as other time to event analyses (eg, time to mortality) will be analyzed using a Cox proportional hazards regression model with a gamma-distributed random intercept for site. We will analyze the ordinal outcome of GOS using a mixed-effects ordinal logistic regression model. The proportional odds assumption will be checked to assess if the relationship between the consecutive outcome levels is the same, and if violated a multinomial logit mixed-effects model will be used.

For the secondary outcome of 90-day in-hospital mortality, a logistic mixed model will be used to conduct non-inferiority and superiority analyses. First, non-inferiority will be assessed using a one-sided test with a significance level of 0.05 and a two-sided 90% confidence interval. The non-inferiority null hypothesis will be rejected if the upper limit of the 90% confidence interval of the estimated absolute risk difference is greater than the non-inferiority margin of 1%. Following testing non-inferiority, we will use two-sided 95% confidence intervals and p-values to assess superiority at the 5% significance level.

## Missing Data

Based on our preliminary data, some missingness of the oxygen exposure or measurements is expected due to inconsistencies in charting, for which we will impute values per our prior analytic approach. Specifically, FiO2 measurements for mechanically ventilated patients will be assumed to remain constant until the patient is extubated or a new setting is recorded. In addition, we will assume that supplemental oxygen will remain constant until the next record (either FiO2, supplemental oxygen, or room air) or for 12 hours. After 12 hours, the patient will be assumed to be on room air. No value of FiO2 was assumed until the first recorded value of a patient’s visit for the first 12 hours. After 12 hours with no oxygen supplementation record, the patient was assumed to be on room air.

## Design Considerations

The intervention will be sequentially rolled out to the 8 enrolled sites (i.e., clusters), switching from control to intervention every 3 months at 8 different time points (**Figure 7**). Each phase will roll-out the intervention in a single site. The control data in the “Pre” phase will be collected retrospectively and thus is not included in the study timeline. The design incorporates a 1-month transition period for training/implementation in each cluster, during which training will be delivered and to allow for full implementation of the intervention. During the transition period, the cluster cannot be considered as either receiving the intervention or the control, and thus patients treated in the emergency department and ICU during that period will not be included in the analysis.

| Site | Pre | | Phase 1 | | | Phase 2 | | | | Phase 3 | | | | Phase 4 | | | | Phase 5 | | | | | Phase 6 | | | | | Phase 7 | | | | Phase 8 | | | |  |  |
| --- | --- | --- | --- | --- | --- | --- | --- | --- | --- | --- | --- | --- | --- | --- | --- | --- | --- | --- | --- | --- | --- | --- | --- | --- | --- | --- | --- | --- | --- | --- | --- | --- | --- | --- | --- | --- | --- |
| 1 |  | |  | |  |  | | | |  | | | |  | | | |  | | | | |  | | | | |  | | | |  | | | |  |  |
| 2 |  | |  | | |  | |  |  |  | | | |  | | | |  | | | | |  | | | | |  | | | |  | | | |  |  |
| 3 |  | |  | | |  | | | |  | |  |  |  | | | |  | | | | |  | | | | |  | | | |  | | | |  |  |
| 4 |  | |  | | |  | | | |  | | | |  | |  |  |  | | | | |  | | | | |  | | | |  | | | |  |  |
| 5 |  | |  | | |  | | | |  | | | |  | | | |  | |  | |  |  | | | | |  | | | |  | | | |  |  |
| 6 |  | |  | | |  | | | |  | | | |  | | | |  | | | | |  | |  | |  |  | | | |  | | | |  |  |
| 7 |  | |  | | |  | | | |  | | | |  | | | |  | | | | |  | | | | |  | |  |  |  | | | |  |  |
| 8 |  | |  | | |  | | | |  | | | |  | | | |  | | | | |  | | | | |  | | | |  | |  |  |  |  |
| *-3* | | *0* | | *3* | | | *6* | | | | *9* | | | | *12* | | | | *15* | | | | | *18* | | | | | *21* | | | | *24* | | | |  |
|  | |  | |  | | |  | | | |  | | | |  | | | |  | | | | |  | | | | | *Time (months)* | | | | | | | |  |
|  | |  | | **Control Period** | | | | | | |  | | | | **Transition** | | | | | |  | | | | | **Intervention Period** | | | | | | | | | | | |

## Figure 7. Stepped wedge randomization scheme

## 9.4 Power

The power calculation is based on the primary outcome of supplemental oxygen-free days (SOFD) within 28 days.^39^ Patients dying within this period are assigned a SOFD score of 0. Preliminary data was used to estimate a mean outcome in the control condition of 15.6; the standard deviation (SD) of the outcome was estimated to be 11.2. We have estimated an intraclass correlation coefficient (ICC) for this data of ~0.04. Although this estimate is based on data from only three sites, it is consistent with estimates for similar outcome variables, which range from 0.01 to 0.05.^40^ With the full sample of 6000 patients over the course of the study, we are able to detect a difference in mean SOFD between control and intervention conditions of 1.41 days at 80% power and a difference of 1.63 days at 90% power. This sample size corresponds to an approximate accrual rate of 25 patients per month per site, assuming 2.5 years of data collection. With a reduction of 25% in eligible patients (i.e., approximately 19 patients per month per site), we still have 80% power to detect a difference of 1.62 days and 90% power to detect a difference of 1.87 days.

Several assumptions of this power calculation are potentially violated in our setting. ^39^ First, while the calculations assume a normally distributed outcome, preliminary data suggest that this assumption is questionable due to skewness, bimodality, and range restrictions. Second, our design incorporates a washout period during which no patients will contribute data (**Figure 7**), while the calculations assume that every site is contributing an equal number of data points in each study period. Third, there is a wide range of sample sizes available at the participating study sites: between 500 and 1500 trauma ICU patients per year on average are expected, while the calculations assume that each site contributes the same number of patients as all other sites. For these reasons, we conducted simulation studies to verify the results of the power calculations presented above. These simulations used a proportional odds model for the outcome to deal with violations of the normality assumption, removed a fraction of patients during each site’s crossover period corresponding to the washout, and used the relative sizes of each site (in terms of number of patients expected) to address deviations from the equal cluster size assumption. These simulation studies demonstrated comparable or increased power relative to estimates obtained from traditional power calculations (**Table**). The results presented above are therefore from the traditional power calculations to provide a conservative estimate of the study’s power.

| **Mean Difference** | **Monte Carlo** | **HH formula** |
| --- | --- | --- |
| 0.5 | 0.17 | 0.17 |
| 1.0 | 0.50 | 0.52 |
| 1.5 | 0.84 | 0.86 |
| 2.0 | 0.98 | 0.98 |
| 2.5 | 1.00 | 1.00 |
| 3.0 | 1.00 | 1.00 |

**Table. Power for testing superiority of intervention**

# ADVERSE EVENTS

# 10.1 Definition of AEs/SAEs

An adverse event is defined as any untoward medical occurrence in a clinical investigation participant administered an intervention that does not necessarily have to have a causal relationship with this treatment. An adverse event therefore can be any unfavorable and unintended sign, symptom, or disease temporally associated with the use of an intervention, whether or not the incident is considered related to the intervention.

A serious adverse event (SAE) is defined as any unexpected and untoward medical occurrence that meets any of the following criteria:

1. Results in death
2. Is life threatening (defined as an event in which the participant was at risk of death at the time of the event and NOT an event that hypothetically might have caused death if it would have been more severe)
3. Requires inpatient hospitalization
4. Prolongs an existing hospitalization
5. Results in persistent or significant disability or incapacity
6. Important medical event that requires an intervention to prevent any of 1-5 above.

The overall PI and local site PIs will be responsible for overseeing the safety of this trial on a daily basis. They will be available any time for questions from the clinical team, who will also be monitoring the patients continuously for adverse events and serious adverse events. Serious and unexpected adverse events potentially associated with study interventions will be recorded in a case report form in the study record and promptly reported to the IRB.

# 10.2 Reporting of Adverse Events

In order to ensure proper and timely reporting of all adverse events, there will be a clear communication plan for all study personnel to follow. Serious and unexpected adverse events potentially associated with study interventions will be reported to the PI within 72 hours of occurrence and recorded in a case report form in the study record. The overall study PI will, in turn, report all SAEs potentially related to study procedures to the cIRB, Independent Safety Monitor, and DoD Scientific Officer within 7 calendar days of occurrence in accordance with IRB policy.

Determination of association with the study intervention will be at the direction of the attending physician. In other words, we will only report on the above listed SAEs if the attending physician believes the study intervention was contributory. All attending physicians will receive training about the intervention and serious adverse event identification and reporting. As we are seeking critically injured patients who will have expected complications of their presenting condition, SAEs that the attending physician determines are unrelated to the study intervention (e.g., due to their overall critical condition) will not be reported.

## REFERENCES CITED

1. Leverve XM. To cope with oxygen: a long and still tumultuous story for life. *Crit Care Med* 2008;36:637-638.
2. Damiani E, et al. Arterial hyperoxia and mortality in critically ill patients: a systematic review and meta- analysis. *Crit Care* 2014;18:711.
3. Panwar R, et al. Current oxygenation practice in ventilated patients: an observational cohort study. *Anaesth Intensive Care* 2013;41:505-514.
4. Suzuki S, et al. Current oxygen management in mechanically ventilated patients: a prospective observational cohort study*. J Crit Care* 2013;28:647-654.
5. Rachmale S, et al. Practice of excessive F(IO(2)) and effect on pulmonary outcomes in mechanically ventilated patients with acute lung injury. *Respir Care* 2012;57:1887-1893.
6. Parke RL, et al .. Oxygen therapy in non-intubated adult intensive care patients: a point prevalence study. *Crit Care Resusc* 2013;15:287-293.
7. Iscoe S, et al. Supplementary oxygen for nonhypoxemic patients: O2 much of a good thing? *Crit Care* 2011;15:305.
8. Panwar R, et al. Conservative oxygen therapy in mechanically ventilated patients. *Crit Care Med* 2014;42:e630-631.
9. de Graaff AE, et al. Clinicians’ response to hyperoxia in ventilated patients in a Dutch ICU depends on the level of FiO2. *Intensive Care Med* 2011;37:46-51.
10. Girardis M, et al. Effort of conservative vs conventional oxygen therapy on mortality among patients in an intensive care unit. The Oxygen-ICU randomized clinical trial. *JAMA* 2016;316:1583-1589.
11. de Jonge E, et al. Association between administered oxygen, arterial partial oxygen pressure and mortality in mechanically ventilated intensive care unit patients. *Crit Care* 2008;12:R156.
12. Pannu SR. Too Much oxygen: hyperoxia and oxygen management in mechanically ventilated patients. *Semin Respir Crit Care Med* 2016;37:16-22.
13. Kallet RH, et al. Should oxygen therapy be tightly regulated to minimize hyperoxia in critically ill patients? *Respir Care* 2016;61:801-817.
14. Hafner S, et al. Hyperoxia in intensive care, emergency, and peri-operative medicine: Dr. Jekyll or Mr. Hyde? A 2015 update. *Ann Intensive Care* 2015;5:42.
15. Helmerhorst HJF, et al. Association between arterial hyperoxia and outcome in subsets of critical illness: a systematic review, meta-analysis, and meta-regression of cohort studies. *Crit Care Med* 2015;43:1508-1519.
16. Austin MA, et al. Effect of high flow oxygen on mortality in chronic obstructive pulmonary disease patients in the prehospital setting: randomized controlled trial. *BMJ* 2010;341:c5462.
17. Chi JH, et al. Prehospital hypoxia affects outcome in patients with traumatic brain injury: a prospective multicenter study. *J Trauma* 2006;61:1134-1141.
18. Panwar R, et al. Conservative versus liberal oxygenation targets for mechanically ventilated patients. A pilot multicenter randomized controlled trial. *Am J Resp Crit Care Med*. 2016;193:43-51.
19. ICU-ROX Investigators et al. Conservative Oxygen Therapy during Mechanical Ventilation in the ICU. *N Engl J Med*. 2019; in press (PMID: 31613432).
20. Schmidt B, et al. Effects of targeting higher vs lower arterial oxygen saturations on death or disability in extremely preterm infants: a randomized clinical trial. *JAMA* 2013;309:2111-2120.
21. Stenson BJ, et al. Oxygen saturation and outcomes in preterm infants. *N Engl J Med* 2013;368:2094-2104.
22. Carlo WA, et al. Target ranges of oxygen saturation in extremely preterm infants. *N Engl J Med* 2010;362:1959-1969.
23. Stockinger ZT, et al. Prehospital supplemental oxygen in trauma patients: its efficacy and implications for military medical care. *Mil Med* 2004;169:609-612.
24. Meyhoff CS, et al. Effect of high perioperative oxygen fraction on surgical site infection and pulmonary complications after abdominal surgery: the PROXI randomized clinical trial. *JAMA* 2009;302:1543-1550.
25. Stub D, et al. Air versus oxygen in ST-segment-elevation myocardial infarction. *Circulation.* 2015;131:2143-2150.
26. Suzuki S, et al. Conservative oxygen therapy in mechanically ventilated patients: a prospective observational cohort study. *J Crit Care* 2013;28:647-654.
27. Suzuki S, et al. Conservative oxygen therapy in mechanically ventilated patients: a pilot before-and-after trial. *Crit Care Med* 2014;42:1414-1422.
28. Eastwood GM, et al. Intensive care clinicians’ opinion of conservative oxygen therapy (SpO2 90-92%) for mechanically ventilated patients. *Aust Crit Care* 2014;27:120-125.
29. Helmerhorst HJ, et al. Self-reported attitudes versus actual practice of oxygen therapy by ICU physicians and nurses. *Ann Intensive Care* 2014;4:23.
30. Helmerhorst HJ, et al. Effectiveness and clinical outcomes of a two-step implementation of conservative oxygen targets in critically ill patients: a before and after trial. *Crit Care Med* 2016;44:554-563.
31. The Acute Respiratory Distress Syndrome Network. Ventilation with lower tidal volumes as compared with traditional tidal volumes for acute lung injury and the acute respiratory distress syndrome. *N Engl J Med* 2000;342:1301-1308.
32. Rachmale S, et al. Practice of excessive FiO2 and effect on pulmonary outcomes in mechanically ventilated patients with acute lung injury. *Respir Care* 2012;57:1887-1893.
33. Mohr CJ, Keenan S. Prolonged field care working group position paper: operational context for prolonged field care. *J Spec Oper Med* 2015;15:78-80.
34. Ball JA, Keenan S. Prolonged field care working group position paper: prolonged field care capabilities. *J Spec Oper Med* 2015;15:76-77.
35. Gangidine MM, et al. System design verification for closed loop control of oxygenation with concentrator integration. *Mil Med* 2016;181:177-183.
36. Baker DW, et al. Criteria for waiver of informed consent for quality improvement research. *JAMA Intern Med* 2015;175:142-143.
37. McKinney RE Jr, et al. Use of altered informed consent in pragmatic clinical research. *Clin Trials* 2015;12:494-502.
38. Douin DJ, Schauer SG, Anderson EL, Jones J, DeSanto K, Cunningham C, Bebarta VS, Ginde AA. Systematic review of oxygenation and clinical outcomes to inform oxygen targets in critically ill trauma patients. *J Trauma Acute Care Surg* 2019;87:961-977.
39. Hussey, M. A., & Hughes, J. P. (2007). Design and analysis of stepped wedge cluster randomized trials. *Contemporary clinical trials*, *28*(2), 182-191.
40. Campbell, M.K., Fayers, P.M. and Grimshaw, J.M., 2005. Determinants of the intracluster correlation coefficient in cluster randomized trials: the case of implementation research. *Clinical Trials*, *2*(2), pp.99-107.
