# Supplementary material for: A multicenter cluster randomized, stepped wedge implementation trial for targeted normoxia in critically ill trauma patients: study protocol and statistical analysis plan for the Strategy to Avoid Excessive Oxygen (SAVE-O2) trial
Source: Trials. 2021 Nov 8;22:784. doi: 10.1186/s13063-021-05688-6 (PMC8574946; doi:10.1186/s13063-021-05688-6)
Supplement: Supplementary file 3 — Additional file 3: Appendix 3: Statistical Analysis Plan [file 13063_2021_5688_MOESM3_ESM.pdf]

Statistical Analysis Plan

**TRIAL FULL TITLE**	Strategy to Avoid Excessive Oxygen (SAVE-O2) for Critically Ill Trauma Patients
**SAP VERSION**	1.0
**SAP VERSION DATE**	2/3/2021
**TRIAL STATISTICIAN**	John D. Rice, PhD
**Protocol Version (SAP associated with)**	1.0
**TRIAL PRINCIPAL INVESTIGATOR**	Adit A. Ginde, MD, MPH
**SAP AUTHOR(s)**	John D. Rice, PhD

1 SAP Signatures

I give my approval for the attached SAP entitled SAVE-O2 dated 2/3/2021.

Statistician (Author)

Name: John D. Rice, PhD

Signature: ______________________________

Date: 2/3/2021______________________

Principal Investigator

Name: Adit A. Ginde, MD, MPH

Signature:______________________________

Date: 2/3/2021______________________

2 Table of Contents

1 SAP Signatures 2
2 Table of Contents 3
3 Abbreviations and Definitions 4
4 Study Objectives and Endpoints 5
4.1 Study Objectives 5
4.2 Endpoints 5
4.2.1 Primary Endpoint 5
4.2.2 Secondary Clinical Endpoints 5
4.2.3 Secondary Oxygenation Endpoints 6
4.2.4 Covariates 6
5 Study Methods 7
5.1 General Study Design and Plan 7
Figure 1.Schematic of the stepped wedge, cluster randomized trial design 7
5.2 Inclusion-Exclusion Criteria and General Study Population 7
5.2.1 Inclusion Criteria 8
5.2.2 Exclusion Criteria 8
5.3 Randomization 8
6 Power and Sample Size 8
Figure 2. Stepped wedge randomization scheme 9
7 General Analysis Considerations 9
7.1 Covariates and Subgroups 10
7.2 Differential treatment effects and subgroup analyses 11
7.3 Missing Data 11
7.4 Interim Analyses and Data Monitoring 11
8 Summary of Study Data 11
8.1 Tables and Figures 12
9 Statistical Analysis Methods 12
9.1 Primary Endpoint 13
9.2 Secondary Endpoints 13
9.2.1 Continuous Outcomes 14
9.2.2 Dichotomous Outcomes 14
9.2.3 Time-to-event Outcomes 15
9.2.4 Ordinal Outcomes 15
Works Cited 16

3 Abbreviations and Definitions

ARDS = Acute Respiratory Distress Syndrome
CCATT = Critical Care Air Transport Team
CCC = Clinical Coordinating Center
CCCRP = Combat Casualty Care Research Program
CI = Confidence Interval
COMIRB = Colorado Multiple Institutional Review Board
CPG = Clinical Practice Guidelines
CT = Computed Tomography
DCC = Data Coordinating Center
DoD = Department of Defense
ED = Emergency Department
EFIC = Exception from Informed Consent
EHR = Electronic Health Record
ERC = En-route care
FDA = U.S. Food and Drug Administration
FiO2 = Fraction of Inspired Oxygen
GOS = Glasgow Outcome Score
HFD90 = Hospital Free Days to Day 90
HRPO = Human Research Protection Office
ICC = Intraclass Correlation Coefficient
ICU = Intensive Care Unit
IRB = Institutional Review Board
JTS = Joint Trauma System
JWMRP = Joint Warfighter Medical Research Program
LAR = Legal Authorized Representative
SOFD= Supplemental Oxygen Free Days
PaO2 = Partial Pressure of Oxygen in Arterial Blood
PETAL = Prevention and Early Treatment of Acute Lung Injury
PFC = Prolonged Field Care
PHI = Protected Health Information
PI = Principal Investigator
SpO2 = Oxygen saturation via pulse oximetry
TCCC = Tactical Combat Casualty Care
USAMRMC = US Army Medical Research and Materiel Command
USSOCOM = United States Special Operations Command

VFD28 = Ventilator Free Days to Day 28

4 Study Objectives and Endpoints

4.1 Study Objectives

The objective is to determine the effectiveness of a multimodal educational intervention to reduce supplemental oxygen use in critically injured patients. We hypothesize that a multimodal educational intervention to limit use of excessive supplemental oxygen will reduce exposure to hyperoxia and safely lower the use of concentrated oxygen.

4.2 Endpoints

In both the pre- and post-implementation phases, we will follow patients for a period of 90 days or until hospital discharge to determine the effect of the interventions on important primary and secondary clinical outcomes. Both pre-implementation and post-implementation data will be collected retrospectively from all sites. Thus, the start of the study will be the same at all sites, though the timing of implementation of the intervention will vary based on the randomization. The individuals enrolled at the end of the last phase of the study will continue to be followed for 90 days to obtain follow-up on their clinical outcomes.

4.2.1 Primary Endpoint

The primary endpoint is supplemental oxygen free days (SOFD) to day 28, defined as number of days alive and not on supplemental oxygen during the index hospitalization. This outcome has a range of -1 days (worst outcome) to 28 days (best outcome). Patients who die during the first 28 days of hospitalization are assigned -1 SOFD. Patients who are discharged alive within 28 days without supplemental oxygen or with prior volume of baseline (pre-hospitalization) home oxygen are assumed to remain alive without additional supplemental oxygen to day 28. Patients who are discharged alive on supplemental oxygen, if above any prior baseline home oxygen therapy, are assumed to remain alive and have additional supplemental oxygen to day 28.

4.2.2 Secondary Clinical Endpoints

All secondary outcomes are assessed throughout hospitalization. There is no follow-up post discharge, and so last observed value will be carried forward as appropriate.

1. **Hospital-free days to day 90 (HFD90)**, defined as the number of days alive and outside the hospital between the initial ED visit and 90 days later. This outcome is a composite of in-hospital mortality and length of hospital stay and has a range of 0 days (worst outcome) to 90 days (best outcome). Patients who die in the hospital within the 90-day observation period are assigned a value of -1 for hospital-free days, while those who remain in the hospital for the entire study are assigned a value of 0 hospital-free days. Patients discharged to any location prior to day 90 are assumed to survive to day 90 and are assigned [90 – hospital length of stay] hospital-free days.
2. **In-hospital mortality to day 90**, defined as a dichotomous vital status (survived or died) at hospital discharge or day 90, whichever is first
3. **Time to mortality to day 90**, based on vital status and date of death and censored at hospital discharge or day 90, whichever is first

4. **Ventilator Free Days to day 28 (VFD28)**: VFD depends on both duration of ventilation and mortality through study day 28. In participants who survive 28 days, VFD is defined as 28 minus duration of ventilation. Duration of ventilation is counted from the first study day of mechanical ventilation (MV) through the last day of MV provided the last day is prior to day 28. Otherwise, it is counted from the first study day of MV through day 28. For participants discharged with MV (e.g., to LTAC facility) prior to day 28, the patient will be assumed to require MV through day 28 (zero VFD will be assigned). Participants discharged from the hospital prior to day 28 (but not to home) on unassisted breathing will be assumed to remain on unassisted breathing through day 28. Isolated periods of ventilation briefer than 24 hours for surgical procedures and ventilation solely for sleep disordered breathing do not count towards duration of ventilation. In participants who never require assisted breathing, duration of ventilation is zero (28 VFD assigned). Participants who do not survive 28 days will be assigned -1 VFD.
5. **Time to Room Air**, defined as the time from hospital presentation to the first episode of no supplemental oxygen (FiO2 0.21 or room air), censored at discharge or death.
6. **Glasgow Outcome Score (GOS),** as assessed by chart review at hospital discharge with one of the following five categories: Death, Persistent vegetative state, Severe disability, Moderate disability, Low disability
7. **Discharge Disposition**, defined as home (return to prior level of care) or facility (e.g, acute rehab, skilled nursing facility)

4.2.3 Secondary Oxygenation Endpoints

1. **Amount of supplemental oxygen administered**, defined as total estimated oxygen volume while in the ICU after hospital arrival
2. **Duration of time on normoxia protocol target**, defined as SpO2 90-96% or receiving no supplemental oxygen (FiO2 0.21 or room air) while in the ICU
3. **Proportion of participants receiving high levels of supplemental oxygen** (FiO2 >0.40 or >4 liters per minute) for >2 hours while in the ICU. This excludes time in the operating room.
4. **Duration of time receiving high levels of supplemental oxygen** (FiO2 >0.40 or >4 liters per minute) while in the ICU
5. **Duration of time receiving no supplemental oxygen** (FiO2 0.21 or room air) while in the ICU
6. **Incidence of hypoxic events** (SpO2 <88%) while in the ICU
7. **Duration of hypoxic events** (SpO2 <88%) while in the ICU
8. **Incidence of hyperoxic events** (SpO2 >96%) while in the ICU
9. **Duration of hyperoxic events** (SpO2 >96%) while in the ICU

4.2.4 Covariates

To adjust for baseline characteristics and severity of illness, we will collect demographics (age, sex, race/ethnicity, payer), military status, mechanism of injury, injury severity score, shock index, Elixhauser comorbidity index, cigarette smoking status, body mass index, and COVID-19 status. We will use standard, validated methods to extract these data, as described in the study protocol. Operational definitions are provided in the data dictionary (Appendix A).

5 Study Methods

5.1 General Study Design and Plan

This study will be a multicenter cluster randomized, stepped wedge implementation trial of a multimodal educational intervention to target normoxia in adult trauma patients admitted to the intensive care unit (ICU). Randomization to receive the intervention occurs sequentially at the hospital level for a phased roll-out of the enhanced education and informatics tools to better achieve the consensus-based normoxia target. This well-accepted stepped wedge trial approach is a one-way crossover trial where all sites will ultimately implement the intervention, and the timing of the intervention implementation is randomly ordered. The intervention will be sequentially rolled out to the 8 enrolling sites (i.e., clusters), switching from control to intervention every 3 months at 8 different time points (**Figure 1**).

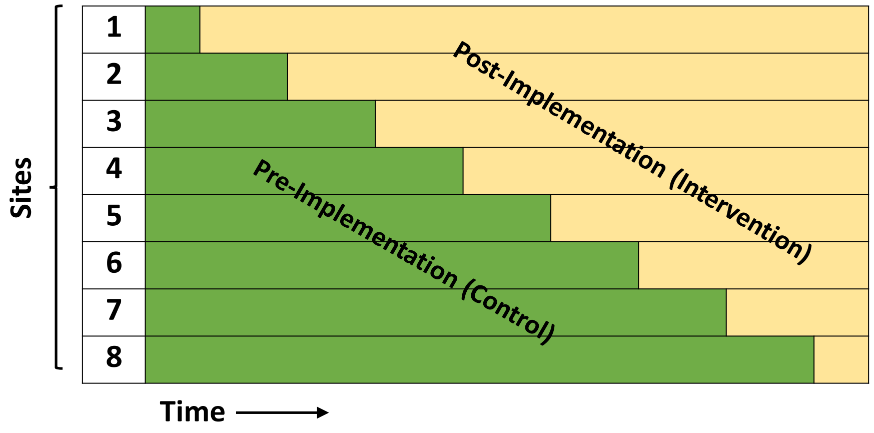

Figure 1.Schematic of the stepped wedge, cluster randomized trial design

We define the consensus-based normoxia target based on thresholds defined in our prior work, which included experts from our proposed sites—oxygen saturation (SpO2) 90-96% and when available, arterial oxygen pressure (PaO2) 60-100 mmHg. The intervention will start in the emergency department upon patient arrival to the hospital and the duration of the intervention period will be the duration of the index ICU stay. The goal is to improve oxygenation to >90% of eligible patient-hours spent in the desired normoxia range, excluding time without supplemental oxygen or time on FiO2 100% and below the normoxia range. Each hospital site will contribute pre-implementation (control) and post-implementation (intervention) data, with the start of the consensus-based intervention period defined by the randomized timing in the stepped wedge design.

The design incorporates a 1-month transition period for staff education/implementation at each hospital, during which training will be delivered and to allow for full implementation of the education and consensus-based intervention. The CCC and overall PIs will provide standardized education and materials to local research teams for site implementation. During the transition period, the cluster cannot be considered as either receiving the structured usual care intervention or the unstructured usual care control, and thus patients treated in the emergency department and ICU during that period will not be enrolled or included in the analysis.

5.2 Inclusion-Exclusion Criteria and General Study Population

Hospitals eligible for participation have endorsed the consensus-based normoxia recommendation of SpO2 90-96% for critically ill trauma patients but currently have no specific plans or resources to promote this oxygenation target during the phased implementation of the enhanced educational intervention. We will define the target population as acutely injured patients who meet criteria for entry into the state/national trauma registry and are admitted to the surgical/trauma ICU within 24 hours of hospital arrival. We anticipate that patients will present primarily to the participating trauma centers emergency departments, although we will include patients transferred to the participating emergency department from another hospital. We will exclude transferred patients who are not admitted through the emergency department. Additional exclusion criteria are age <18 years, prisoners, or known pregnancy (we expect that all female patients of childbearing age will receive a pregnancy test per usual care as part of standard protocols). We will include both mechanically ventilated and non-mechanically ventilated patients. We will pre-define specific trauma subgroups, including hemorrhagic shock, mechanical ventilation, injury severity/mechanism, and traumatic brain injury.

5.2.1 Inclusion Criteria

1. Acutely injured patients who meet the criteria for entry into the state or national trauma registry
2. Admission to surgical/trauma ICU within 24 hours of hospital arrival

5.2.2 Exclusion Criteria

1. Age <18 years
2. Prisoners
3. Known pregnancy
4. Transferred patients not admitted through the emergency department

5.3 Randomization

In this stepped wedge design, each Trauma site will be randomized to one of 8 possible crossover time points: this will be done by randomly permuting the list of sites participating in the trauma trial. The sites will cross over in the order thereby generated (see **Figure 1**). To accommodate sites that may be unable to initiate the intervention in the first time period of the study due to local logistical reasons, the randomization may be constrained as follows: One of the 6-7 sites that are able to go first will be randomly chosen and assigned to cross over at the first time period. Then the remaining sites, including those unable to go first, would be permuted as described above and assigned to cross over at times 2-8.

6 Power and Sample Size

The power calculation is based on the primary outcome of supplemental oxygen-free days (SOFD) within 28 days [1]. Preliminary data was used to estimate a mean outcome in the control condition of 15.5; the standard deviation (SD) of the outcome was estimated to be 11.3. We have estimated an intraclass correlation coefficient (ICC) for this data of ~0.04. Although this estimate is based on data from only three sites, it is consistent with estimates for similar outcome variables, which range from 0.01 to 0.05 [2]. With the full sample of 6000 patients over the course of the study, we are able to detect a difference in mean SOFD between control and intervention conditions of 1.42 days at 80% power and a difference of 1.64 days at 90% power. This sample size corresponds to an approximate accrual rate of 25 patients per month per site, assuming 2.5 years of data collection. With a reduction of 25% in

eligible patients (i.e., approximately 19 patients per month per site), we still have 80% power to detect a difference of 1.63 days and 90% power to detect a difference of 1.89 days.

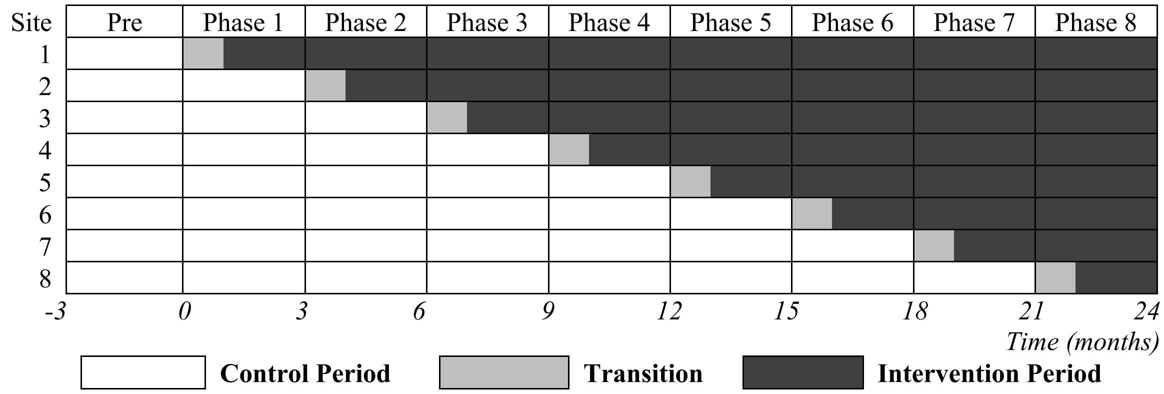

Figure 2. Stepped wedge randomization scheme

Several assumptions of this power calculation are potentially violated in our setting [1]. First, while the calculations assume a normally distributed outcome, preliminary data suggest that this assumption is questionable due to skewness, bimodality, and range restrictions. Second, our design incorporates a partial washout period in each site's crossover phase during which no patients will contribute data (**Figure 2**), while the calculations assume that every site is contributing an equal number of patients in each study period. Third, there is a wide range of sample sizes available at the participating study sites: between 500 and 1500 trauma ICU patients per year on average are expected, while the calculations assume that each site contributes the same number of patients as all other sites.

For these reasons, we conducted simulation studies to verify the results of the power calculations presented above. These simulations used a proportional odds model for the outcome to deal with violations of the normality assumption, removed a fraction of patients during each site's crossover period corresponding to the washout, and used the relative sizes of each site (in terms of number of patients expected) to address deviations from the equal cluster size assumption. These simulation studies demonstrated comparable or increased power relative to estimates obtained from traditional power calculations (**Table**). The results presented above are therefore drawn from the traditional power calculations to provide a conservative estimate of the study's power. An additional feature of the planned analysis that contributes to making the power calculations presented here conservative is covariate adjustment, which should increase power. [3] [4]

Mean Difference	Monte Carlo	HH formula
0.5	0.16	0.17
1.0	0.50	0.52
1.5	0.84	0.86
2.0	0.97	0.98
2.5	1.00	1.00
3.0	1.00	1.00

Table. Power for testing superiority of intervention

7 General Analysis Considerations

As an overall approach, we will analyze primary and secondary endpoints using a mixed

effects modeling framework, with specific distributions chosen depending on the type of outcome (e.g., binary, count, ordinal, time-to-event). The primary analysis will be the effect of the treatment condition on supplemental oxygen-free days. With the stepped-wedge design of the study, the intervention is implemented at different times at the different hospitals; therefore, a fixed effect for time will be included to adjust for possible temporal trends. Data collected during the 1-month transition phase for each site will not be used in the primary analysis. Each subject will have a time variable equal to an integer 1-9 corresponding to the 3-month interval in which he or she was admitted. Time will be treated categorically in the model, i.e., will comprise 8 regression coefficients, one for each study period after the first, which will be treated as the reference category. We will account for clustering of patients within sites by including a random intercept term in all mixed models; marginal models will instead use a robust covariance estimator. Robust (empirical) covariance estimators will also be considered for mixed models where misspecification is suspected. For all hypotheses, unless explicitly stated otherwise, significance tests will be two-sided at the 5% significance level. There is a single primary endpoint, and we will not adjust for multiple comparisons. Analyses will be conducted in SAS or R.

7.1 Covariates and Subgroups

To improve precision, we will adjust final models for pre-selected patient-level covariates:

1. Age
2. Sex
    - Male
    - Female
    - Other
3. Race/ethnicity
    - Non-Hispanic white
    - Non-Hispanic black
    - Hispanic
    - Asian
    - Other
4. Payer/insurance type
    - Private/Commercial
    - Medicaid
    - Medicare
    - Self-pay/uninsured
    - Other
5. Elixhauser Comorbidity Index [5]
6. Mechanism of injury
    - Blunt
    - Penetrating
7. Injury Severity Score
8. Cigarette smoking status
    - Current smoker
    - Former smoker
    - Never smoker
9. Body mass index
10. COVID-19 status

Continuous covariates will be modeled with polynomial terms or splines to capture any

nonlinear effects and ensure adequate control of confounding variables in this cluster-randomized study. AIC [6] will be used to determine the appropriate complexity of nonlinear terms. Because we anticipate adequate sample size and event rates, no other variable selection will be performed for the models.

7.2 Differential treatment effects and subgroup analyses

Subgroup analyses will be conducted by including interactions in regression models between the treatment condition and the specific covariate of interest (e.g., trauma subgroups, categories of injury severity score). A significant coefficient estimate for the interaction term(s) will suggest that the effect of treatment varies according to the value of that covariate. In this event, separate estimates of the treatment effect will be reported for each level of the interacting covariate. Significant interactions between the treatment effect and continuous covariates will be summarized graphically in addition to reporting treatment effects for a representative selection of values for the continuous covariate.

7.3 Missing Data

Based on our preliminary data, some missingness of the oxygen exposure or measurements is expected due to inconsistencies in charting, for which we will impute values. Specifically, FiO2 measurements for mechanically ventilated patients will be assumed to remain constant until the patient is extubated or a new setting is recorded. In addition, for non-mechanically ventilated patients, we will assume that supplemental oxygen will remain constant until the next record (either FiO2, supplemental oxygen, or room air) for 12 hours. After 12 hours, the patient will be assumed to be on room air unless otherwise noted. No value of FiO2 will be assumed until the first recorded value of a patient's visit for the first 12 hours. After 12 hours with no oxygen supplementation record, the patient will be assumed to be on room air. Missing covariates will be handled using multiple imputation by chained equations (MICE), with 5 imputed data sets generated, analyzed, and results combined according to the standard rules for MI. [7]

7.4 Interim Analyses and Data Monitoring

Because the focus of the intervention is on achieving oxygenation targets (particularly reducing exposure to hyperoxia), and summary implementation and clinical data will be provided quarterly, formal interim analyses will not be required.

8 Summary of Study Data

All continuous variables will be summarized using the following descriptive statistics: *n* (non-missing sample size), mean/median, and standard deviation/interquartile range (IQR). The frequency and percentages of observed levels will be reported for all categorical measures. All summary tables will be structured with a column for the full sample as well as each intervention condition and will be annotated with the total sample size relevant to that table. Note that due to the stepped-wedge design, different sites will contribute different sample sizes to each of the experimental conditions. Therefore, we will also generate summary tables by site.

8.1 Tables and Figures

The analysis report will include the following tables and figures.

1. **Figure**. CONSORT Diagram
2. **Table**. Patient Characteristics
    - Overall
    - Stratified by treatment condition

    a. Demographics (age, sex, race/ethnicity, payer/insurance type)
    b. Comorbidities (Elixhauser Comorbidity Index, cigarette smoking status, BMI, COVID-19 status)
    c. Injury (initial SpO2 and FiO2, vital signs and shock index, mode of arrival, mechanism of injury, Injury Severity Score)
3. **Table**. Primary endpoint (SOFD)
    - Overall
    - Stratified by treatment condition

    a. Mean (SD) among survivors
    b. In-hospital mortality within 28 days (frequency/proportion)
    c. Frequency/Proportion alive with 0 SOFD
    d. Frequency/Proportion alive with 28 SOFD
4. **Figure**. Histogram of SOFD
    - Stratified by treatment condition
5. **Table**. Secondary endpoints
    - Overall
    - Stratified by treatment condition

    a. Clinical endpoints
        i. Hospital-free days to day 90
        ii. In-hospital mortality to day 90
        iii. Ventilator Free Days to day 28
        iv. Time to Room Air
        v. Glasgow Outcome Score
        vi. Discharge Disposition
    b. Oxygenation endpoints
        i. Amount of supplemental oxygen administered
        ii. Duration of time on normoxia protocol target
        iii. Proportion of participants receiving high levels of supplemental oxygen
        iv. Duration of time receiving high levels of supplemental oxygen while in the ICU
        v. Duration of time receiving no supplemental oxygen
        vi. Incidence of hypoxic events
        vii. Duration of hypoxic events
        viii. Incidence of hyperoxic events
        ix. Duration of hyperoxic events
6. **Figure**. Time to mortality
    - Kaplan-Meier curves
    - Stratified by treatment condition

9 Statistical Analysis Methods

All analyses are intention to treat, that is, all eligible patients will be included in the analyses specified. All hypothesis testing will be two-tailed at the 5% level of significance. There is a single composite endpoint, and no adjustment will be made for multiple comparisons with respect to secondary endpoints and analyses; appropriate caution will therefore be used in interpreting the results of hypothesis testing for these analyses. Analysis will be based on all available data. There will be no missingness on the primary outcome due to the way it is measured. For other outcomes, we will use an inverse probability weighting approach (probability for missing the outcome), followed by a sensitivity complete case analysis. Missing covariates will be handled as described above.

9.1 Primary Endpoint

We will analyze the primary endpoint, supplemental oxygen-free days (SOFD) using the generalized linear mixed modeling (GLMM) framework to account for correlation within sites. This approach will produce conditional estimates. Secondarily, to produce marginal estimates, the generalized estimating equations (GEE) approach will be used. Summary tables for the outcome will be produced by site and treatment condition. Graphical summaries will include histograms, also stratified by site and treatment condition. The linear modeling framework allows for the direct estimation of the effect of the intervention on the conditional mean of the outcome, so our primary analysis will be undertaken within this framework. Although evidence from preliminary studies suggests that residuals for this outcome are not normally distributed, both simulation studies undertaken during the planning phase of this trial as well as those reported in the literature [8] [9] show considerable robustness of the linear mixed modeling framework to violation of distributional assumptions. In the event of more substantial violations of modeling assumptions, we will assess whether other distributions provide a better fit. If there is a larger proportion of patients who experience mortality than expected, zero-inflated mixed models may be considered. We will also consider alternative modeling approaches such as cumulative logit mixed models treating the response as an ordinal outcome to avoid more parametric assumptions. Comparisons between outcome distributions will be made based on AIC, with models with substantially lower AIC receiving further consideration [6]. The systematic part of the model will be

$$Y_{ijk} = \mu + \beta_j + X_{ij}\theta + \mathbf{z}'_{ijk}\boldsymbol{\gamma} + \alpha_i + e_{ijk}$$

- Outcome Y_{ijk} is SOFD for patient k at time j in cluster i
- β_j is a fixed effect for time interval, with $\beta_0 = 0$ for identifiability
- X_{ij} is a treatment indicator in cluster i at time j, equal to 0 for control and 1 for intervention
- θ is the treatment effect, mean difference between the outcome in patients in the intervention and control conditions
- $\mathbf{z}_{ijk}$ is a vector of covariates to be adjusted for, with $\boldsymbol{\gamma}$ the corresponding regression coefficients
- α_i is a random effect for cluster, assumed to be normally distributed. (This term is not explicitly modeled in the GEE framework.)
- e_{ijk} is residual error, also assumed to be normally distributed.

The clinical objective, to determine if the intervention affects SOFD, will be assessed by testing the significance of the coefficient for intervention condition, θ.

9.2 Secondary Endpoints

Some secondary endpoints will require normalization for length of stay in hospital or ICU. **Duration** endpoints (e.g., time spent hypoxic) will be analyzed using a GLMM with continuous outcome distribution (e.g., normal, gamma), log link and offset for log(length of time exposed). This approach will yield parameter estimates that are log ratios of proportions of time spent in a given state. **Incidence** endpoints will be analyzed using a GLMM with count outcome distribution (e.g., Poisson, negative binomial) and offset for log(length of time exposed). This approach will yield parameter estimates that are log rate ratios. Both approaches will appropriately account for patients spending differing lengths of time under study. Specific statistical models for individual secondary outcomes are detailed below.

9.2.1 Continuous Outcomes

Continuous secondary outcomes, including hospital-free days, ventilator-free days, oxygen volume required, and number of hypoxic/hyperoxic events will be analyzed similarly to the primary endpoint, with a linear mixed modeling approach. As with the primary endpoint, we will consider alternative modeling approaches such as cumulative logit mixed models to avoid more parametric assumptions while addressing the ordinal nature of the outcomes, with model comparisons being made on the basis of AIC. Comparisons between intervention and control conditions will be made by significance testing of the coefficient θ as estimated from the model; this parameter represents the mean difference between the outcome in patients in the intervention and control conditions, adjusting for covariates.

9.2.2 Dichotomous Outcomes

Dichotomous outcomes such as whether a patient requires high flow oxygen (>4 L/min or FiO2 >40%) will be analyzed using a logistic mixed model. The model for these analyses will be

$$\log\frac{P(Y_{ijk}=1)}{P(Y_{ijk}=0)} = \beta_0 + \beta_j + X_{ij}\theta + \mathbf{z}'_{ijk}\boldsymbol{\gamma} + \alpha_i$$

- Outcome Y_{ijk} is equal to 1 if patient *k* at time *j* in cluster *i* experienced the event (e.g., required high flow oxygen) and 0 otherwise
- β_j is a fixed effect for time interval, with $\beta_0 = 0$ for identifiability
- X_{ij} is a treatment indicator in cluster *i* at time *j*, equal to 0 for control and 1 for intervention
- θ is the treatment effect, log odds ratio between intervention and control conditions
- $\mathbf{z}_{ijk}$ is a vector of covariates to be adjusted for, with $\boldsymbol{\gamma}$ the corresponding regression coefficients
- α_i is a random effect for cluster, assumed to be normally distributed. (This term is not explicitly modeled in the GEE framework.)

Comparisons between intervention and control conditions will be made by significance testing of the coefficient θ as estimated from the model.

For the secondary outcome of 90-day in-hospital mortality, a logistic mixed model will be used to conduct non-inferiority and superiority analyses. First, non-inferiority will be assessed using a one-sided test with a significance level of 0.05 and a two-sided 90% confidence interval. The non-inferiority null hypothesis will be rejected if the upper limit of the 90% confidence interval of the estimated absolute risk difference is greater than the non-inferiority margin of 1%. Following testing non-inferiority, we will use two-sided 95% confidence intervals and p-values to assess superiority at the 5% significance level.

9.2.3 Time-to-event Outcomes

The time-to-room air outcome, as well as other time to event analyses (e.g., mortality) will be analyzed using a Cox proportional hazards regression model with a gamma-distributed random intercept for site. Time zero will be determined as time of arrival. Kaplan-Meier plots will also be used to examine the unadjusted distribution of time to the event of interest and make comparisons between treatment conditions. Plots by site will also be produced. The regression model for these analyses will be

$$\log h_{ijk}(t) = \log h_0(t) + \beta_j + X_{ij}\theta + \mathbf{z}'_{ijk}\boldsymbol{\gamma} + \log \alpha_i$$

- $h_{ijk}(t)$ is the hazard for the event of interest at time *t* for patient *k* at time j in cluster *i*
- β_j is a fixed effect for time interval, with $\beta_0 = 0$ for identifiability
- X_{ij} is a treatment indicator in cluster *i* at time j, equal to 0 for control and 1 for intervention
- θ is the treatment effect, log hazard ratio between intervention and control conditions
- $\mathbf{z}_{ijk}$ is a vector of covariates to be adjusted for, with $\boldsymbol{\gamma}$ the corresponding regression coefficients
- α_i is a random effect for cluster, assumed to be gamma distributed. (This term is not explicitly modeled in the GEE framework.)

Comparisons between intervention and control conditions will be made by significance testing of the coefficient θ as estimated from the model.

9.2.4 Ordinal Outcomes

We will analyze the ordinal outcome of Glasgow Outcome Score (GOS) using a mixed-effects ordinal logistic regression model. This approach will also be used for other outcomes (including SOFD) in the event that the prescribed analytic approach is found to be inadequate. The model for these analyses will be

$$\log \frac{P(Y_{ijk} \le l)}{P(Y_{ijk} > l)} = \beta_0 + \beta_j + X_{ij}\theta + \mathbf{z}'_{ijk}\boldsymbol{\gamma} + \alpha_i, l = 1, \dots, L-1$$

- Outcome Y_{ijk} is equal to *l* for patient *k* at time j in cluster *i* as follows:
  - Death ($l = 1$)
  - Persistent vegetative state ($l = 2$)
  - Severe disability ($l = 3$)
  - Moderate disability ($l = 4$)
  - Low disability ($l = 5$)
- β_j is a fixed effect for time interval, with $\beta_0 = 0$ for identifiability
- X_{ij} is a treatment indicator in cluster *i* at time j, equal to 0 for control and 1 for intervention
- θ is the treatment effect, log cumulative odds ratio between intervention and control conditions
- $\mathbf{z}_{ijk}$ is a vector of covariates to be adjusted for, with $\boldsymbol{\gamma}$ the corresponding regression coefficients
- α_i is a random effect for cluster, assumed to be normally distributed. (This term is not explicitly modeled in the GEE framework.)

The proportional odds assumption will be checked to assess if the relationship between the consecutive outcome levels is the same, and if violated a multinomial logit or partially proportional odds mixed-effects model will be used.

Works Cited

[1] M. A. Hussey and J. P. Hughes, "Design and analysis of stepped wedge cluster randomized trials," *Contemporary Clinical Trials,* vol. 28, no. 2, pp. 182-191, 2007.

[2] M. K. Campbell, P. M. Fayers and J. M. Grimshaw, "Determinants of the intracluster correlation coefficient in cluster randomized trials: the case of implementation research," *Clinical Trials,* vol. 2, no. 2, pp. 99-107, 2005.

[3] C. B. Kahan, V. Jairath, C. J. Doré and T. P. Morris, "The risks and rewards of covariate adjustment in randomized trials: an assessment of 12 outcomes from 8 studies," *Trials,* vol. 15, 2014.

[4] E. L. Turner, P. Perel, T. Clayton, P. Edwards, A. V. Hernández, I. Roberts, H. Shakur, E. W. Steyerberg and CRASH trial collaborators, "Covariate adjustment increased power in randomized controlled trials: an example in traumatic brain injury," *Journal of Clinical Epidemiology,* vol. 65, no. 5, pp. 474-481, 2012.

[5] A. Elixhauser, C. Steiner, D. R. Harris and R. M. Coffey, "Comorbidity measures for use with administrative data," *Medical Care,* vol. 36, no. 1, p. 8–27, 1998.

[6] H. Akaike, "A new look at the statistical model identification," *IEEE Transactions on Automatic Control,* vol. 19, no. 6, p. 716–723, 1974.

[7] S. van Buuren and K. Groothuis-Oudshoorn, "mice: Multivariate Imputation by Chained Equations in R," *Journal of Statistical Software,* vol. 45, no. 3, pp. 1-67, 2011.

[8] H. Schielzeth, N. J. Dingemanse, S. Nakagawa, D. F. Westneat, H. Allegue, C. Teplitsky, D. Réale, N. A. Dochtermann, L. Z. Garamszegi and Y. G. Araya-Ajoy, "Robustness of linear mixed-effects models to violations of distributional assumptions," *Methods in Ecology and Evolution,* 2020.

[9] A. F. Schmidt and C. Finan, "Linear regression and the normality assumption," *Journal of Clinical Epidemiology,* vol. 98, pp. 146-151, 2018.
